# Molecular basis of resistance to the microtubule-depolymerizing antitumor compound plocabulin

**DOI:** 10.1038/s41598-018-26736-3

**Published:** 2018-06-05

**Authors:** Areti Pantazopoulou, Carlos María Galmarini, Miguel A. Peñalva

**Affiliations:** 1 Department of Cellular and Molecular Biology, Centro de Investigaciones Biológicas, CSIC, Madrid, Spain; 2 Departamento de Biología Celular y Farmacogenómica, Pharma Mar S.A., Colmenar Viejo, Madrid, Spain; 3 Present Address: Department of Molecular Genetics and Cell Biology, University of Chicago, 920 East 58th Street, Chicago, IL 60637 USA

## Abstract

Plocabulin (PM060184) is a microtubule depolymerizing agent with potent antiproliferative activity undergoing phase II clinical trials for the treatment of solid tumors. Plocabulin shows antifungal activity virtually abolishing growth of the filamentous fungus *Aspergillus nidulans*. *A*. *nidulans* hyphae depend both on mitotic and interphase microtubules, as human cells. Here, we exploited the *A*. *nidulans* genetic amenability to gain insight into the mechanism of action of plocabulin. By combining mutations in the two *A*. *nidulans* β-tubulin isotypes we obtained a plocabulin-insensitive strain, showing that β-tubulin is the only molecular target of plocabulin in fungal cells. From a genetic screen, we recovered five mutants that show plocabulin resistance but do not carry mutations in β-tubulin. Resistance mutations resulted in amino acid substitutions in (1) two subunits of the eukaryotic translation initiation factor eIF2B activating the General Amino Acid Control, (2) TIM44, an essential component of the inner mitochondrial membrane translocase, (3) two transcription factors of the binuclear zinc cluster family potentially interfering with the uptake or efflux of plocabulin. Given the conservation of some of the identified proteins and their respective cellular functions in the tumor environment, our results pinpoint candidates to be tested as potential biomarkers for determination of drug efficiency.

## Introduction

**Figure 1:**
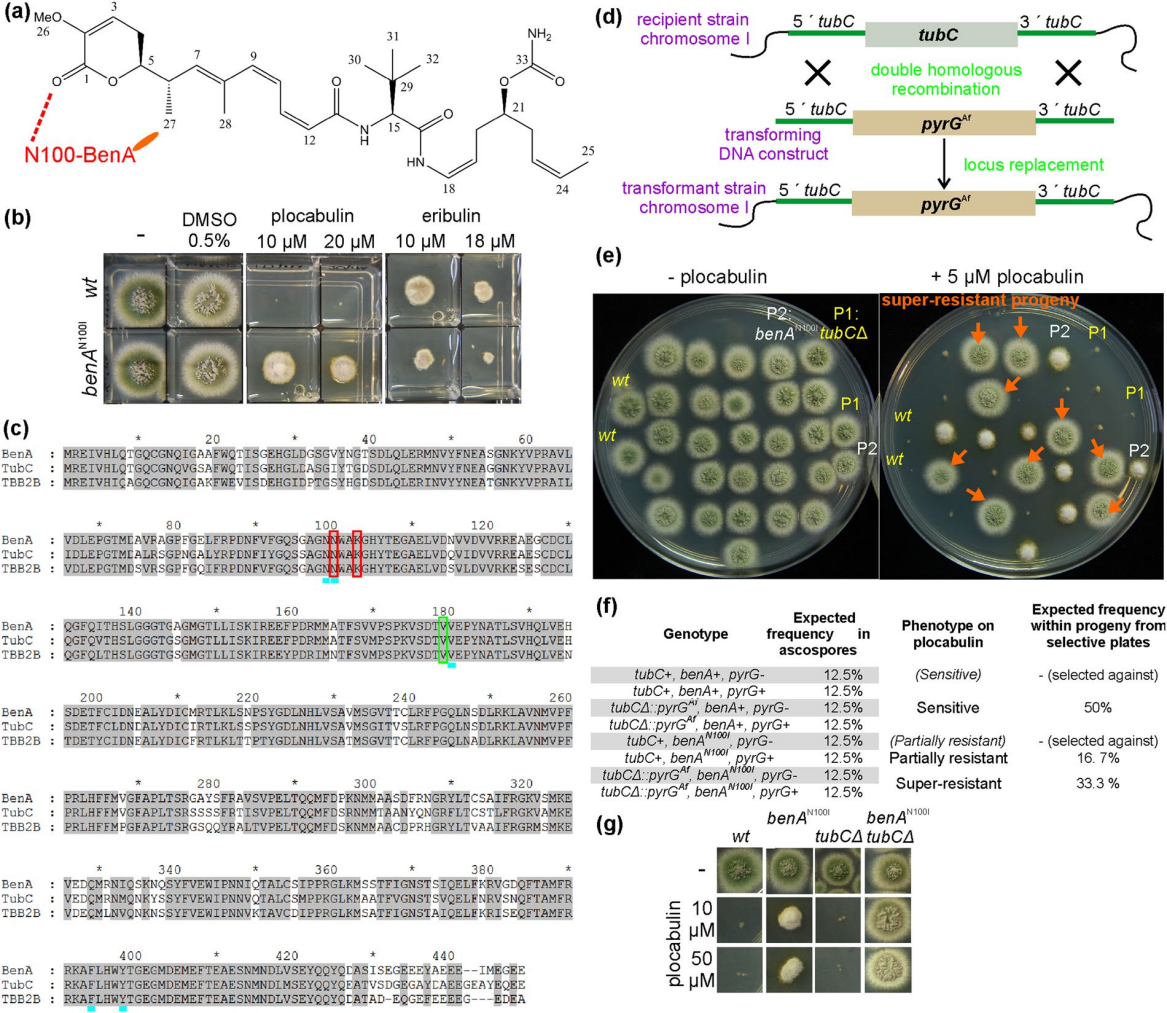
The sole molecular target of plocabulin in *A*. *nidulans* is β-tubulin. (**a**) Chemical structure of plocabulin. Based on the structure of tubulin in complex with plocabulin [ref.^2^], Asn100 in *A*. *nidulans* β-tubulin, BenA, would form a hydrogen (H) bond (dashed line) with plocabulin position 1 and would contribute to hydrophobic interactions (ellipse) with position 27. (**b**) Growth test of a *wt* and the *benA*^N100I^ strain. Consistent with Asn100 forming part of the PM06018 binding site, *benA*^N100I^ confers resistance to plocabulin, albeit partial. *benA*^N100I^ shows hypersensitivity to eribulin, a drug with a different binding site on β-tubulin. (**c**) Alignment of the two *A*. *nidulans* β-tubulin isotypes, BenA and TubC, with *Bos taurus* β-tubulin TUBB2B. (BenA: AN1182, TubC:AN6838; TUBB2B: *B*. *taurus* TBB2B_BOVIN, Q6B856). Both β-tubulin isotypes conserve the amino acid residues that form the plocabulin binding site, i.e. Asn99/Asn100/Lys103/Val179/Val180/Phe394/Tyr398. Red box: aa forming hydrogen bonds with plocabulin position 1, Green box: aa forming hydrogen bond with plocabulin position 13, Blue low dash: aa participating in hydrophobic interactions with plocabulin position 27^2^. (**d**) Strategy used to delete the *tubC* ORF: The *Aspergillus fumigatus pyrG* (*pyrG*^*Af*^) complements pyrimidine auxotrophy of *pyrG*^−^ *A*. *nidulans*. This allows the selection of double homologous recombination that mediates replacement of *tubC* by *pyrG*^*Af*^ (*tubC*Δ::*pyrG*^Af^) when an appropriated DNA construct is used for transformation. (**e**) Growth on plocabulin of progeny from a cross between *tubC*Δ (P1) and *benA*^N100I^ (P2): ≈42% (n = 24) displayed plocabulin sensitivity, like the *wt* and *tubC*Δ strains, ≈21% showed partial resistance like *benA*^N100I^ and ≈37% showed insensitivity to plocabulin (indicated by orange arrows) unlike either parental. (**f**) Frequency of the super-resistant class was similar to the expected for the double *benA*^N100I^ *tubC*Δ mutant (note that selective plates did not contain pyrimidines, see also text). Genotyping indeed verified that super-resistant strains were *benA*^N100I^ *tubC*Δ. (G) The double *benA*^N100I^ *tubC*Δ mutant shows super-resistance to 10 and 50 µM plocabulin, indicating that at these concentrations the only direct target of plocabulin relevant to fungal growth is β-tubulin.

Plocabulin (PM060184, PM184) (Fig. 1a) is a microtubule (MT) depolymerizing agent currently undergoing phase II clinical trials for the treatment of solid tumors. Initially isolated from the sponge *Lithoplocamia lithistoides*, the compound is currently produced by total synthesis^1^. Plocabulin binds to β-tubulin, sharing its binding site with maytansine and rhizoxin (the “maytansine-site”)^2,3^. Plocabulin binding to β-tubulin leads to a decrease of MT dynamic instability and to MT depolymerization in a concentration-dependent manner^4^. The drug inhibits migration of interphase cells and induces a prometaphasic arrest in mitotic cells, resulting either in the induction of caspase-dependent apoptosis or in the formation of multinucleated, polyploid interphase-like cells^4^. Plocabulin shows potent antitumor activity in tumor xenograft models, including those tumors overexpressing p-glycoprotein^4^, and has demonstrated vascular-disrupting activity^5^. Thus, plocabulin is a promising anticancer drug.

The haploid filamentous fungus *Aspergillus nidulans* is an established genetic model. Unlike the prototypic fungal genetic model *Saccharomyces cerevisiae*, *A*. *nidulans* hyphal cells require MTs not only for enacting mitosis and driving nuclear movement^6^, but also for accomplishing long-distance transport of endosomes and peroxisomes^7,8^ and for efficiently delivering secretory carriers to exocytic sites, in collaboration with actin cables^9,10^. Thus, the roles of MTs in *A*. *nidulans* resemble those in mammalian cells^11–13^. This dependence on MT transport is probably imposed by the large intracellular distances (in the order of 0.1 mm) between apex-proximal and distal regions of the tubular hyphal tip cells^11^. It is thus unsurprising that the field of MT research owes important advances, such as the characterization of the first eukaryotic genes for α, β, and γ-tubulins^14–16^, to genetic studies carried out with this fungus.

We have previously shown that plocabulin exhibits antifungal activity, compromising *A*. *nidulans* colony formation at concentrations >5 µM^3^ (Fig. 1b). Addition of plocabulin to growing hyphae expressing GFP-tagged α-tubulin (TubA-GFP) results in MT depolymerization^3^ (see also Results), indicating that a common mechanism of action (MoA) underlies both the antifungal (against *A*. *nidulans*) and anti-proliferative (against human tumor cell lines) activities displayed by the drug. This suggested that, by using the powerful genetic tools available for *A*. *nidulans*, it would be possible to gain insight into the MoA of plocabulin and the cellular potential for drug resistance. As a proof of concept we had previously isolated from a random genetic screen plocabulin-resistant *A*. *nidulans* mutants that constituted *in vivo* evidence for the existence of a novel plocabulin specific binding site on β-tubulin. These strains carried mutations leading to the Asn100Ile substitution in the major β-tubulin BenA [reported in]^3^, a substitution also shown to confer resistance to rhizoxin^17^. The substitution did not have any effect on growth on medium without plocabulin, strongly indicating that Asn100Ile would affect a putative plocabulin binding site on β-tubulin, rather than general MT structure or dynamics^3^. Notably the crystal structures of tubulin bound to plocabulin, rhizoxin and maytansin were consistent with these conclusions, showing that Asn100 is indeed part of the β-tubulin binding site that contacts a common pharmacophore on these compounds^2^.

Although Asn100 in BenA is critical for plocabulin binding, *A*. *nidulans* carrying *benA*^*N100I*^ is only partially resistant to the drug^3^ (Fig. 1b), suggesting that intracellular targets other than BenA would contribute to plocabulin antifungal activity. By combining directed genetic manipulations with an unbiased genetic screen coupled to next generation sequencing (NGS) and genetic mapping, we investigated (1) whether plocabulin has additional intracellular targets other than β-tubulin in *A*. *nidulans* and (2) the landscape of genes that have the potential, when mutated, to confer plocabulin resistance on *A*. *nidulans*. We show that β-tubulin is the sole direct intracellular target of plocabulin relevant to fungal survival and growth. This negates the hypothetical existence of secondary intracellular targets contributing to drug activity. We then establish a causal relationship between plocabulin partial resistance and mutations in (1) two subunits of the eukaryotic translation initiation factor eIF2B, (2) TIM44, a conserved and essential component of the translocase of the inner mitochondrial membrane, and (3) two DNA binding domain-containing proteins of the zinc binuclear cluster family.

## Results

### Remaining sensitivity of the *benA*^N100I^ strain to plocabulin is due to the expression of a second β-tubulin isotype

Asn100Ile in BenA eliminates a hydrogen bond between the carboxamide group of Asn100 and the carbonyl group at position 1 of plocabulin and probably perturbs hydrophobic interactions between the methyl group at position 27 of the drug and the BenA pocket including Asn100^2^ ^2^ (Fig. 1a). Thus, BenA^N100I^ directly affects drug binding, thereby conferring resistance to plocabulin at concentrations ranging from 5–50 µM (Fig. 1b,g)^3^. However, resistance is only partial, as manifested by reduced colony diameter and conidiation (production of asexual spores) displayed by the *benA*^*N100I*^ strain growing on plocabulin-containing plates (Fig. 1b). Consistent with the fact that plocabulin and rhizoxin share their binding site on β-tubulin^2,17^, *benA*^*N100I*^ shows cross-resistance to rhizoxin (not shown). In contrast, this mutant is hypersensitive to the MT depolymerizing agents benomyl (see below), and eribulin (Fig. 1b) that bind to different domains in β-tubulin^18^. These findings suggest that *benA*^*N100I*^ substitution changes accessibility to the neighboring benomyl and vinca binding sites. Hypersensitivity to other anti-MT agents combined with rhizoxin resistance could represent a biomarker for plocabulin resistance mutations in β-tubulin. Anti-MT agents with a different binding site might represent an effective therapy in such cases.

Partial resistance of *benA*^*N100I*^ to plocabulin might be due to additional cellular target(s). *A*. *nidulans* has a second β-tubulin, TubC, sharing ~85% amino acid sequence identity with BenA. While BenA, expressed during vegetative growth and conidiation, is essential, TubC, expressed mainly during conidiation, is dispensable for growth^19–22^. Nevertheless, these β-tubulin genes are interchangeable after promoter swapping^19^. Notably, conidiation on benomyl of strains with partial benomyl resistance-conferring *benA* mutations improves when *tubC* is ablated^21^, indicating that both β-tubulins are benomyl targets and that BenA can form both vegetative and conidiation microtubules when TubC is absent. TubC might also be a target for plocabulin, responsible for at least part of the *benA*^*N100I*^ residual plocabulin sensitivity, since the amino acid residues forming the plocabulin binding site are conserved in TubC (Fig. 1c). To test this hypothesis, we deleted *tubC* in a *wt* background (Fig. 1d). The resulting *tubC*Δ was as sensitive to plocabulin as the *wt* (Fig. 1e,g). We next crossed *tubC*Δ and *benA*^N100I^ and analyzed how progeny scored for plocabulin resistance. We identified three phenotypic classes: plocabulin-sensitive (resembling *wt* or *tubC*Δ strains), partially resistant (resembling *benA*^N100I^) and “super-resistant”, apparently unaffected by plocabulin (Fig. 1e). The “super-resistant” phenotype appeared with a frequency consistent with that expected for the double mutant *benA*^N100I^ *tubC*Δ (n = 24, Chi-squared = 0.708, df = 2, two-tailed P > 0.5) (Fig. 1f). Indeed, DNA genotyping demonstrated that this plocabulin super-resistant class corresponds to the double mutant progeny. The *benA*^N100I^ *tubC*Δ double mutant was insensitive to plocabulin over a range of concentrations (5–50 µM) (Fig. 1g). We conclude that: (1) plocabulin targets both β-tubulin isotypes in *A*. *nidulans*; (2) Asn100Ile abrogates the “maytansin-site” on BenA; (3) Although mutations affecting the plocabulin binding site on the major β-tubulin confer resistance to the drug, the presence and/or expression levels of minor β-tubulin isotypes can further modulate such resistance; (4) The sole plocabulin intracellular target affecting fungal growth and survival at the drug concentrations used is β-tubulin.

### Mutations in genes other than tubulins confer resistance to plocabulin

**Figure 2:**
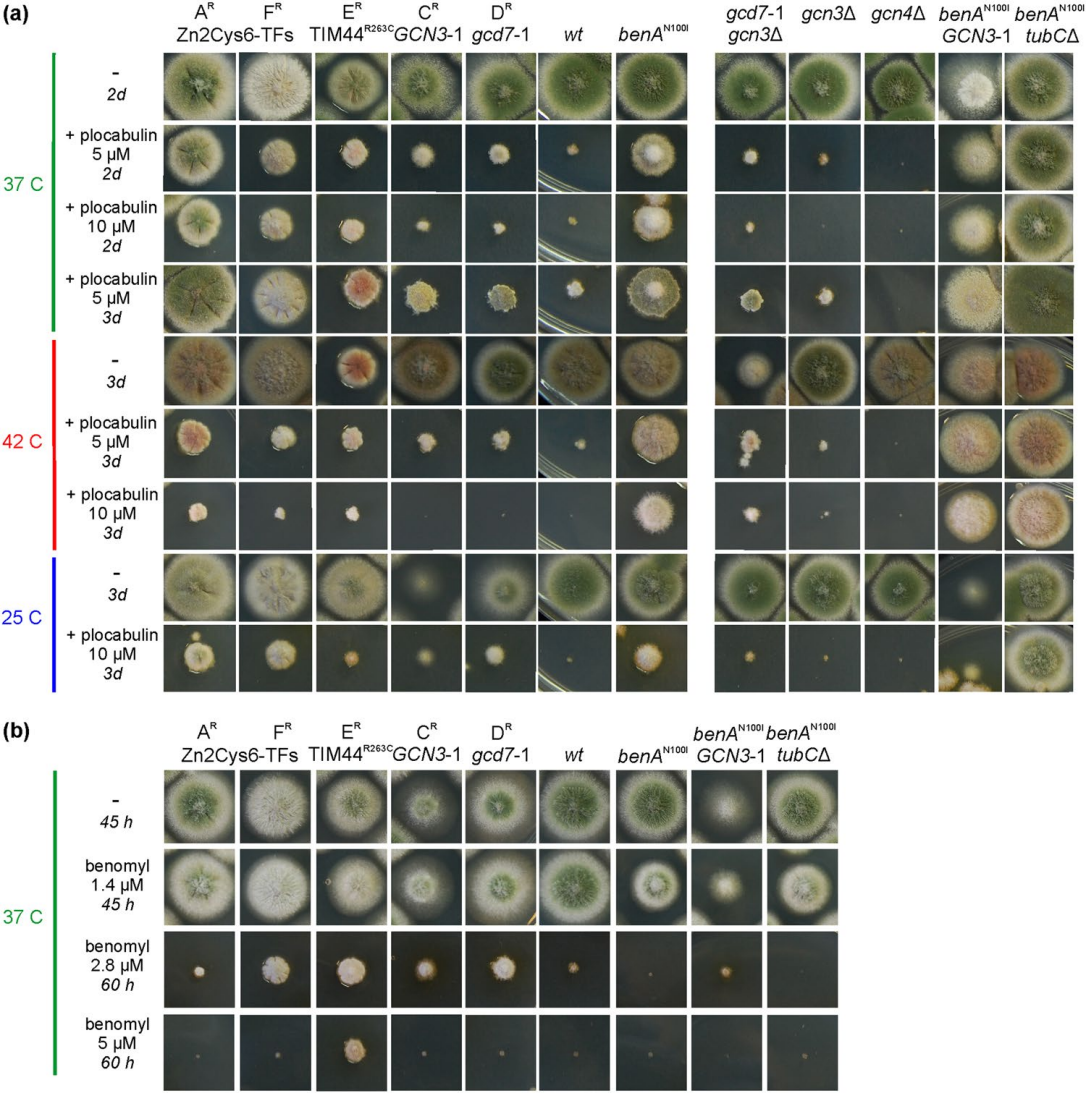
Phenotypic profiling of *A*. *nidulans* plocabulin resistant mutants. (**a**) Growth test of A^R^, F^R^, E^R^, C^R^, D^R^ extra-tubulin mutants selected in a random genetic screen vs. a *wt* strain and the *benA*^*N100I*^-expressing strain (left panel) and of genetically engineered *gcd7*-1 *gcn3*Δ, *gcn3*Δ, *gcn4*Δ, *benA*^*N100I*^ *GCN3*-1, *benA*^*N100I*^ *tubC*Δ mutants strains (right panel) on medium with 5 or 10 µM or no (-) plocabulin. Strains were cultured for 2 or 3 days (*2d* or *3d*) at 37 °C, 25 °C or 42 °C, as indicated. (**b**) Growth of the indicated strains at 37 °C on benomyl (1.4, 2.8 and 5 µM). Colonies were left to grow for the indicated time period. [Strains used: MAD5916 (A^R^), MAD4751(F^R^), MAD6098 (E^R^), MAD6095 (C^R^), MAD5959 (D^R^), MAD5321 (wt), MAD4736 (*benA*^*N100I*^), MAD5656 (*benA*^*N100I*^ *GCN3*-1), MAD5877 (*benA*^*N100I*^ *tubC*Δ), MAD6091 (*gcn4*Δ), MAD5386 (*gcn3*Δ), MAD5948 (*gcd7*-1 *gcn3*Δ), S1 Table].

We then sought for non-tubulin mutations that would confer plocabulin resistance in *A*. *nidulans*. We plated UV light-mutagenized conidia of *A*. *nidulans* on 10 µM plocabulin-containing medium. Resistant colonies were distinguishable over the background of marginally growing colonies (Supplementary Fig. S1). Plocabulin-resistant mutants were classified into five classes, denoted A^R^, C^R^, D^R^, E^R^ and F^R^, according to their degree of resistance and their growth vigor (Fig. 2a) [a sixth resistant class from the same genetic screen, B^R^, had been previously attributed to the *benA*^*N100I*^ mutation]^3^. In all five classes, resistance to plocabulin segregated as a single Mendelian trait in genetic crosses, indicating that resistance was caused by mutation in a single locus (Supplementary Fig. S1). Sequencing of *tubA* and *benA* ruled out the presence of mutations in the major α- and β- tubulin genes.

We checked cross-resistance of the mutants to other anti-MT drugs. All showed resistance to rhizoxin (not shown). Notably, all mutants showed hyper-resistance to benomyl, which contrasts with the hypersensitivity shown by *benA*^*N100I*^ (Fig. 2b). E^R^ showed the highest resistance to benomyl and grew similarly on benomyl and plocabulin. A^R^ showed only marginal benomyl resistance, but the highest plocabulin resistance.

### Effect of resistance mutations on microtubule stability and mitotic progression in the presence of plocabulin

To test whether A^R^, C^R^, D^R^, E^R^ and F^R^ mutations modify the stability of interphase MTs and/or the mitotic defects caused by the drug, we introduced by genetic crosses GFP-TubA to label MTs and HhoA (H1 histone)-mCherry to label chromatin in the five mutant strains (see in ref.^23^ strain LO1915 for a description of these markers and ref.^24^ for the first simultaneous visualization of DNA and spindles in *A*. *nidulans*). The presence of GFP-tagged α-tubulin did not affect resistance to plocabulin (Supplementary Fig. S2).

**Figure 3:**
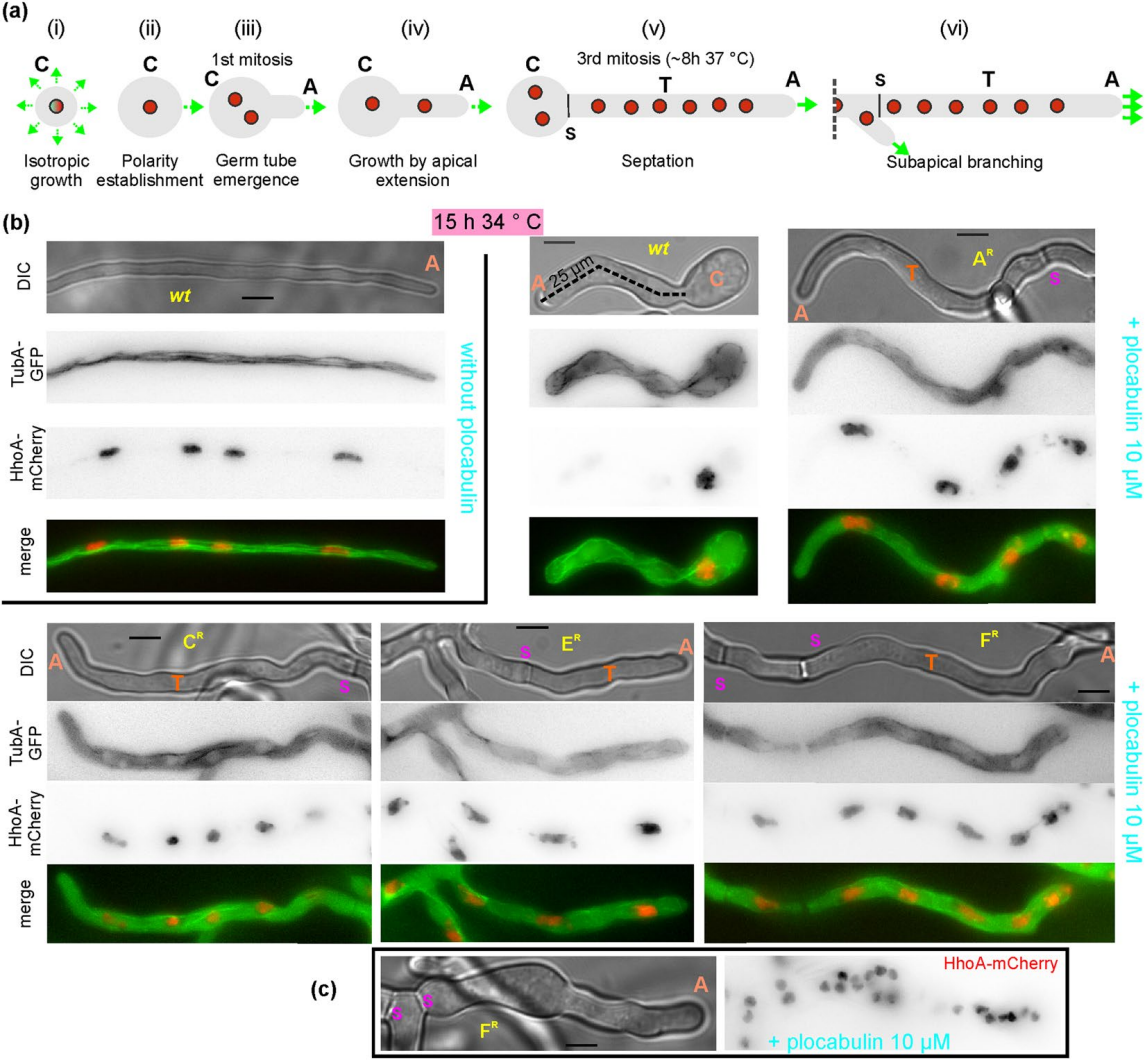
*wt* and mutant strains’ growth, nuclei and microtubules in the presence of plocabulin. (**a**) *A*. *nidulans* asexual spores (conidia) are dormant and uninucleate. Conidia break dormancy soon after their inoculation in liquid medium in microscopy chambers and initiate isotropic growth (i), followed by the establishment of a polarity axis (ii), i.e. the selection of the plasma membrane spot towards which biosynthetic material will be directed leading to germ tube emergence, which takes place around the time when the first mitosis occurs (≈4 h at 37 °C) (iii). Growth continues exclusively by apical extension (iv). The first septum is normally laid after the third mitosis has taken place^25^ (v). As short germlings are being transformed into hyphae (with several multinucleated cellular compartments separated by septae) (vi), their apical extension rate increases, shifting from solely actin-dependent to both actin- and microtubule-dependent transport of exocytic vesicles. (**b**) After ≈15 h, *wt* spores have given rise to hundreds of µm-long hyphae with well discernible HhoA (histone 1)-mCherry-labelled nuclei and GFP-TubA (α tubulin)-labelled microtubules traversing the hyphal tip cell (upper left panel). Resistance mutations do not show any major effect on this growth pattern (not shown). After 15 h in 10 µM plocabulin, *wt* spores only form short germlings, that contain 1 to 2 nuclei and have no septae (upper middle panel), suggesting a block in mitosis. In contrast, resistant mutants form long hyphae that contain multiple nuclei and septae [upper right and lower panels; note that for the resistant strains only the hyphal tip cells are shown, while for the *wt* strain the entire germling can be seen]. Both in the *wt* and the mutants, interphase microtubules are largely depolymerized. (**c**) A large proportion of the F^R^ population displays abnormally high numbers of nuclei and septae per compartment. C: conidiospore; A: apex; T: tip cell; S: septum; The dotted line in (**a**) indicates that only part of the hypha is depicted, green arrows indicate growth direction and rate, red circles represent nuclei. Single wavelength fluorescent microscopy images are inverted contrast maximal projections of z-stacks. All images are at the same magnification. Bar = 5 µm.

Conidia were inoculated into inverted microscopy chambers containing liquid medium and incubated at 34 °C. Uninucleate *A*. *nidulans* conidiospores sediment at the bottom glass, break dormancy, establish a polarity axis and undergo growth by apical extension to form germlings that gradually give rise to hyphae (Fig. 3a). At 8 hours post-inoculation three rounds of mitosis have produced 8 nuclei and the first septum has been formed^25–27^. For both the *wt* and the mutant strains, hyphal length was in the order of hundreds of micrometers and hyphae contained multiple nuclei and well-discernible interphase microtubules at 15 hours post-inoculation (Fig. 3b). Including plocabulin in the growth medium prevented formation of long hyphae in the *wt* strain, which produced instead germlings with very short germ tubes [≈ 22 ± 12 µm (mean ± S.D.) *N* = 77)] and abnormal morphology (Fig. 3b). Most of these germlings had one or two nuclei (Fig. 3b), often containing elongated lumps of chromatin, suggesting defective chromosome segregation and mitotic blockade. Consistently, septae were rarely observed. All resistant mutants progressed beyond the *wt* in the presence of plocabulin (Fig. 3b): E^R^, C^R^ and F^R^ doubled in length the *wt* [≈45 ± 18 µm, ≈54 ± 20 µm and ≈55 ± 35 µm (mean ± S.D.), *N* = 23, 14 and 17; in unpaired *t*-test with Welch’s correction, *P* = <0.0001, <0.0001 and 0.0014, respectively] (measurements in F^R^ were an underestimation because this mutant hyperbranched). A^R^ hyphae grew up to hundreds of µm and also displayed numerous branches. Mitosis was at least partially restored in all resistant mutants, as indicated by the generation of septated hyphae that contained several nuclei per cell (Fig. 3b). Nevertheless, GFP-TubA was largely observed in a cytoplasmic haze, indicating extensive interphase MT depolymerization both in the *wt* and resistant cells, which suggested that the mitotic spindle rather than interphase MTs must be the growth-limiting physiological target of plocabulin in the *wt*.

**Figure 4:**
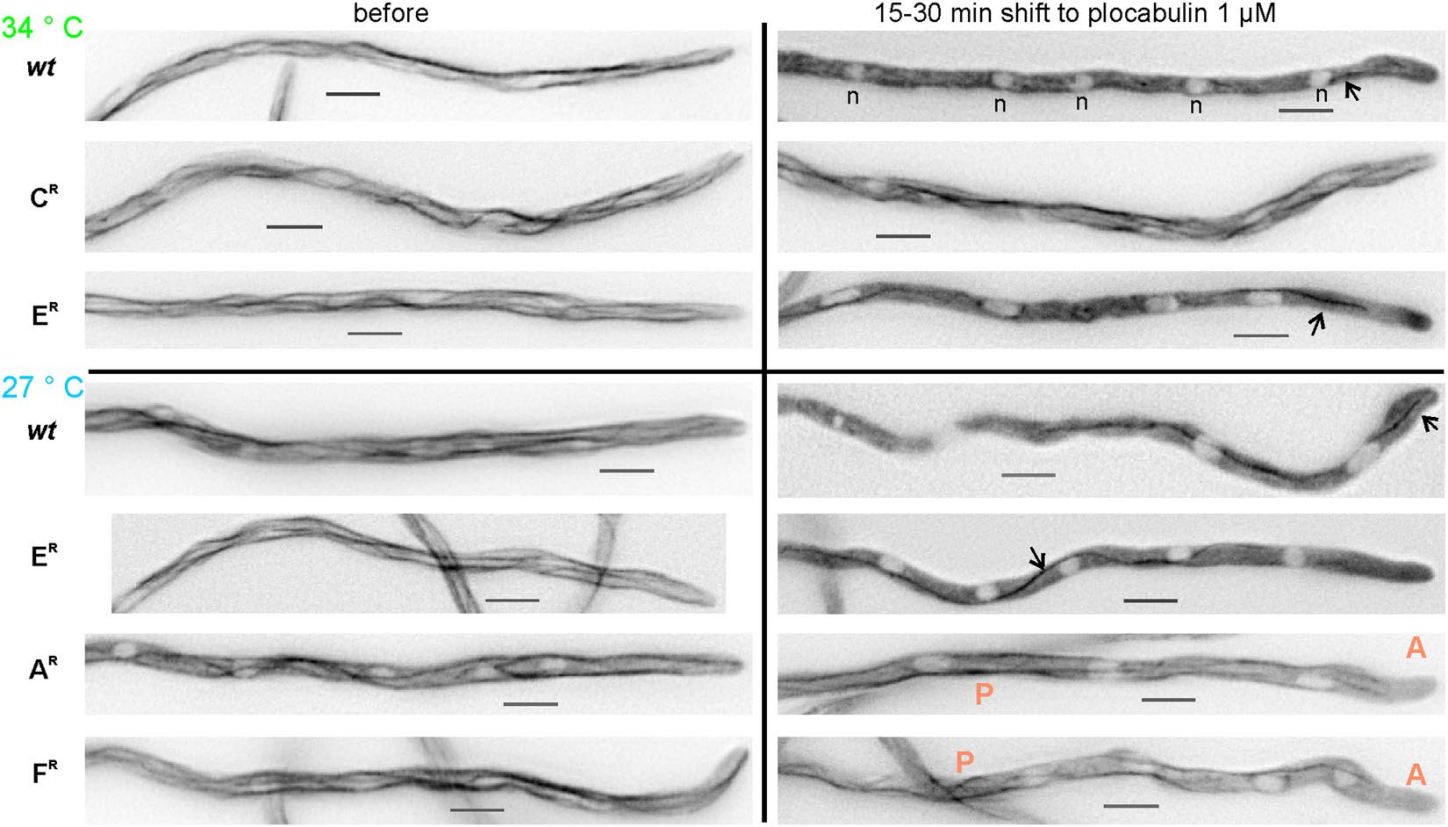
Effect of plocabulin on interphase microtubules of fast-growing plocabulin-resistant hyphae. Conidia from the *wt* and the indicated mutants were left to form fast-growing hyphae (15-18 h) (“Before”). Hyphae were then shifted to 1 µM plocabulin-containing medium and were observed 15 to 30 min after the shift. Note that α-tubulin becomes cytoplasmic in the *wt* and in the E^R^ mutant, with the exception of some microtubule remnants (arrows), possibly reflecting bundles of microtubules stabilized by lateral interactions. Free tubulin is excluded from the nucleoplasm of interphase nuclei (n). MTs are largely resistant to plocabulin in the C^R^ mutant. Interphase MTs are partially resistant in posterior areas (P) of the A^R^ and F^R^ hyphae, but depolymerize close to the hyphal apices (A). Images are sum projections of z-stacks modified with the unsharp mask filter of Metamorph. Bar = 5 µm.

To discern differences, if any, in the stability of interphase MTs, we added 1 µM plocabulin [semi-restrictive in plates]^3^ to fast-growing hyphae pre-cultured without the drug. In the *wt* interphase MTs were rapidly depolymerized at both 27 °C and 34 °C (Fig. 4), resulting in a sharp increase in cytoplasmic GFP-α-tubulin fluorescence. GFP-TubA was also detected in abnormal spindles in metaphase-like nuclei, but these spindles did not elongate, indicating a blockade in metaphase (Supplementary Movie S1)^24,28,29^. In the A^R^ mutant MTs were clearly resistant to the drug at 27 °C, but some depolymerization occurred at the tips such that MTs did not reach the apex (Fig. 4). Mitosis in the A^R^ proceeded beyond metaphase (not shown). The F^R^ mutant showed a similar phenotype, but MTs appeared less resistant to plocabulin than in A^R^ (Fig. 4). In F^R^ mutant hyphae nuclei blocked in prophase/metaphase or proceeding through anaphase were both observed (Supplementary Movie S2). In the C^R^ mutant essentially no depolymerization of interphase MTs was observed (this mutant is cryosensitive and therefore it was tested at 34 °C) (Fig. 4). In the E^R^ mutant, addition of plocabulin at 27 °C resulted in rapid MT depolymerization like in the *wt*, but MTs started recovering after 30–60 min, unlike the *wt*. Because E^R^ resistance is slightly cryosensitive (Fig. 2a), we also tested E^R^ at 34 °C. At this temperature, MTs depolymerized upon drug addition, but this response was not completely homogeneous in the population, indicating marginal resistance. Overall, our results showed that A^R^, C^R^, and F^R^ displayed increased interphase MT stability in the presence of plocabulin, whereas E^R^ had a weak effect. The increased interphase MT resistance in A^R^ and F^R^, as well as the higher resistance displayed by A^R^ MTs compared to F^R^ MTs, correlated with growth on plocabulin-containing plates displayed by these two mutants (Fig. 2a). Notably, the C^R^ mutant displayed the most stable interphase MTs, although on plates it displayed weak resistance, resembling that of the E^R^ strain.

### Mutations in eIF2B that constitutively activate the general amino acid control pathway protect cells from plocabulin

C^R^ and D^R^ mutations conferred moderate-to-low resistance to 5–20 µM plocabulin (Fig. 2a). At 37 °C, C^R^ and D^R^ strains displayed reduced growth and formed sparse mycelium (Fig. 2a). Both were cryosensitive (Fig. 2a). Growth defects were more marked in C^R^. In both mutants reduced growth and cryosensitivity were linked to plocabulin resistance (0 recombinants out of 48 progeny).

**Figure 5:**
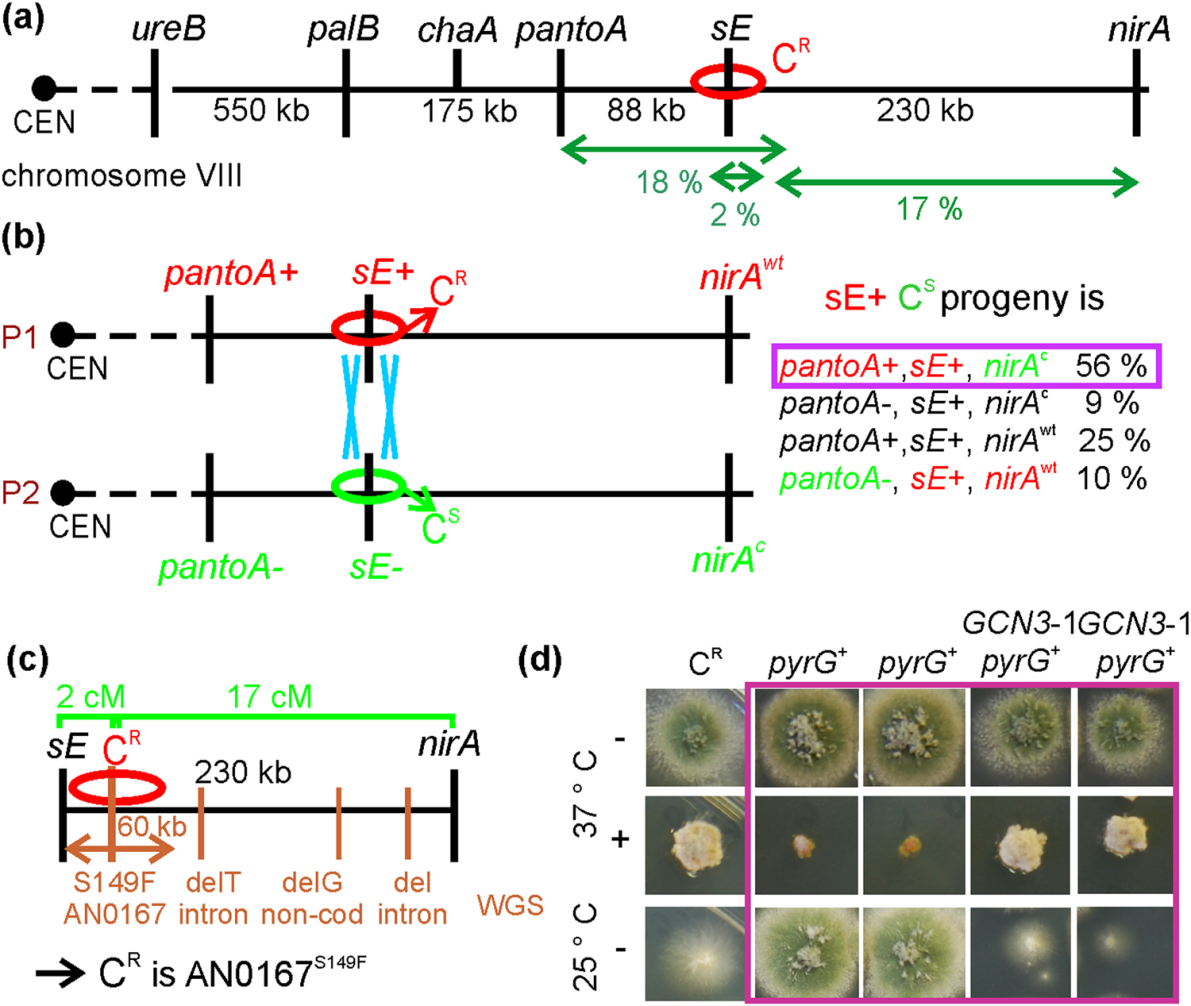
Plocabulin resistance in the C^R^ mutant is conferred by Ser149Phe amino acid substitution in the α subunit of eIF2B, Gcn3. (**a**) Haploidization analysis located the C^R^ mutation on chromosome VIII. Using chromosome VIII markers in meiotic crosses, we detected linkage of the C^R^ mutation with *ureB*3 (P = 0.018, recombination frequency = 36%), *palB*16 (P < 0.0001, recombination frequency = 25%) and *chaA*2 (P < 0.0001, recombination frequency = 19%), suggesting that C^R^ was located centromere-distal to *chaA*. With a subsequent cross we detected 18, 2 and 17% recombination between the C^R^ mutation and *pantoA*, *sE* and *nirA* respectively (progeny n = 96), indicating that the mutation lied between *pantoA* and *nirA*, closely linked to *sE*. (**b**) To determine whether C^R^ is centromere distal or proximal relative to *sE*, we selected progeny in which recombination between the C^R^ and *sE* loci had occurred. In 81% (n = 59) C/*sE* recombinants *pantoA* and *sE* had not recombined, indicating that *sE* lies between *pantoA* and C^R^. In agreement, 65% of the C/*sE* recombinants showed recombination between *sE* and *nirA*, suggesting that C^R^ was between the *sE* [*trxA*^73^] and *nirA*. P1, P2: parental 1 or 2. (**c**) Sequencing of the C^R^ genome (WGS) revealed that the only polymorphism between *sE* and *nirA* close enough to *sE* (<60 kbp) to account for the 2 cM *sE*/C^R^ genetic distance was a double transition within the AN0167 ORF (*GCN3*-1) leading to Asn149Phe in the encoded protein, Gcn3. (**d**) We reconstructed the *GCN3*-1 mutation into an otherwise *wt* strain (*GCN3*-1 *pyrG*^+^) and confirmed that *GCN3*-1 suffices to confer the C^R^-characteristic plocabulin resistance and cryosensitivity. *pyrG*^+^ control transformants that had not incorporated *GCN3*-1 were neither cryosensitive, nor resistant to plocabulin. + and −: medium with/without plocabulin.

We identified the C^R^ mutation by combining genetic mapping with whole genome sequencing (WGS). We constructed a diploid strain combining C^R^ with a tester strain carrying diagnostic markers in each chromosome and, subsequently, promoted chromosomal re-assortment by haploidization^30^. By phenotypic analysis of the haploid segregants we assigned the C^R^ mutation to chromosome VIII (mitotic recombination is very rare in *A*. *nidulans*). We set up meiotic crosses between C^R^ and strains carrying chromosome VIII markers. Linkage analysis suggested the C^R^ mutation was located between the *sE* and *nirA* genes, closely linked to *sE* (Fig. 5a,b). This is a ≈230 kb-long DNA track containing more than 70 candidate open reading frames (ORFs). Sequencing the C^R^ *sE*-*nirA* interval by WGS, we detected two adjacent transitions within the AN0167 ORF (g.493 C > T + g.494 T > C, resulting in the amino acid substitution Ser149Phe), a single *nt* deletion within an intron of AN0149, a single *nt* deletion in an intergenic region and multiple *nt* deletions within the first intron of AN10019 (Supplementary Table S3). The double transition in AN0167 lied at 9 kbp from *sE*, within the range of physical distance expected from recombination frequencies, unlike the non-coding changes, (Fig. 5c) and resulted in a non-conservative amino acid substitution in the encoded protein, strongly indicating that this mutation was causative of the C^R^ plocabulin resistance.

To formally demonstrate this contention, we reconstructed the mutation in a *wt* genetic background. A linear DNA fragment including the C^R^ mutation was used to replace *wt* genomic AN0167 by homologous recombination^31^, after co-transformation of a *pyrG*89 (pyrimidine auxotrophy) strain with mutant AN0167 and *wt pyrG* DNA fragments to facilitate the selection of transformants (Supplementary Fig. S3). We obtained transformants that showed moderate-to-low resistance to plocabulin, displayed the C^R^ characteristic growth defect, were cryosensitive and had replaced *wt* AN0167 by the mutant gene (Fig. 5d), confirming that g.493 C > T + g.494 T > C leading to Ser149Phe in AN0167 was the cause of these phenotypes.

AN0167 is homologue of yeast *GCN3*. We denoted the C^R^ mutant allele *GCN3*-1 (see below). Gcn3 is a subunit of eIF2B, an oligomeric guanine nucleotide exchange factor (GEF) that activates translation initiation factor eIF2, a heterotrimer consisting of subunits α, β and γ. eIF2B promotes recycling of inactive eIF2-GDP released after translation initiation into active eIF2-GTP capable of initiating a new round of translation by delivering methionyl-tRNA to the ribosome^32^. Upon stress such as amino acid starvation, phosphorylation of eIF2α at Ser51 converts eIF2 into a competitive inhibitor of eIF2B, reducing the number of translation initiation complexes and leading to a reduction in general translation^33,34^. This, in turn, elicits global changes in gene expression by facilitating translation of certain mRNAs, such as those of transcription factors Gcn4 (in yeast) and ATF4 (in mammals) that carry short upstream ORFs hindering translation of the main ORF in non-stress conditions^33,34^. This stress response is conserved between fungi and mammals [*G*eneral *A*mino *A*cid *C*ontrol (GAAC) in yeast, *C*ross-*P*athway *C*ontrol (CPC) in *Aspergillus* and *Neurospora* or *I*ntegrative Stress *R*esponse (ISR) in mammals].

eIF2B is a heterodecamer containing two sets of α, β, γ, δ and ε subunits (yeast Gcn3, Gcd7, Gcd1, Gcd2, Gcd6, respectively), where α, β and δ subunits form the regulatory and γ and ε the catalytic sub-complex^35–37^. Tight binding of P-eIF2α into a pocket formed by the regulatory subunits obstructs interaction of eIF2γ (the guanine nucleotide binding subunit in eIF2) with an Asn-Phe motif in eIF2Bε, impeding GDP/GTP exchange on eIF2^38,39^. Genetic studies have shown that *GCN3* encodes a positive regulator of GAAC. Recessive *gcn3* mutations confer sensitivity to amino acid starvation (Gcn^−^ phenotype, *g*eneral *c*ontrol *n*on-inducible). *GCD* genes encode negative-acting subunits, with *gcd* partial loss-of-function mutations leading to constitutive GAAC activation and increased resistance to amino acid starvation (Gcd^−^ phenotype, *g*eneral *c*ontrol *d*erepressed)^33,40,41^.

**Figure 6:**
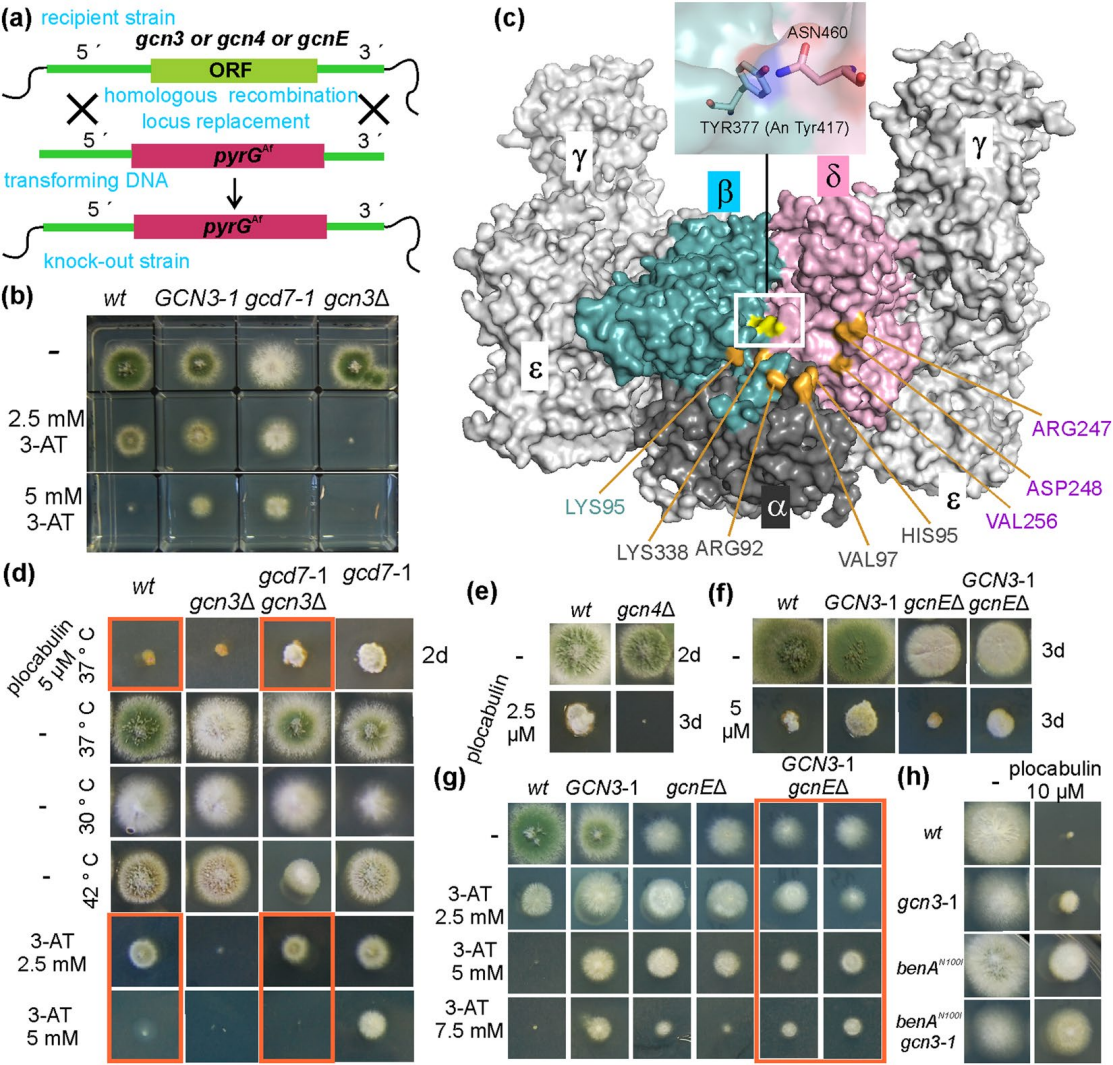
Impact of *GCN3*-1 (C^R^) and *gcd7*-1 (D^R^) on eIF2B and the General Amino Acid Control pathway. (**a**) Strategy used to delete *gcn3*, *gcn4* and *gcnE* ORFs by replacing them with *pyrG*^*Af*^ (Fig. 1D legend). (**b**) Growth of the *wt* (MAD5321) and C^R^ (*GCN3*-1) (MAD6095), D^R^ (*gcd7*-1) (MAD4740) and *gcn3*Δ (MAD5386) mutants on 3-aminotriazol, an inhibitor of histidine biosynthesis that induces the GAAC. (**c**) Surface representation of the *S*. *pombe* eIF2B oligomer [oriented as in Fig. 2, ref.^35^]. Different subunits are indicated with Greek letters. Regulatory subunits α, β and δ form a central cavity thought to accommodate eIF2α. Several residues that are cross-linked to eIF2α in the eIF2B::eIF2α complex are highlighted in orange. Yellow labelling indicates subunit β Tyr377 whose hydroxyl group forms a hydrogen bond with ND2 of Asn460 in δ (see enlargement). β-Tyr377 is the equivalent of Tyr417 in *A*. *nidulans*. *gcd7*-1 (D^R^) results in Tyr417Asn substitution, thereby disrupting the βTyr377 - δAsn460 hydrogen bond and in all likelihood affecting the position of δ Asn460 that contributes to the base of eIF2α binding cavity. (**d**) *gcn3*Δ has a slight effect on the plocabulin resistance conferred by *gcd7*-1, but restores normal (*wt*) resistance to 3-AT, possibly counteracting the effect of *gcd7*-1 on the GAAC pathway (see text) (*gcn3*Δ *gcd7*-1 is MAD5948, *gcd7*-1 is MAD5950, S1 Table). (**e**) *wt* and *gcn4*Δ growing on sub-lethal concentration of plocabulin. *gcn4*Δ results in hypersensitivity to plocabulin. (**f**) Deletion of the histone acetyltransferase *gcnE* impairs growth, but it does not modify the plocabulin resistance conferred by *GCN3*-1. (**g**) Both single *gcnE*Δ and double *gcnE*Δ *GCN3*-1 mutant strains show resistance to 5 and 7.5 mM 3-AT, where *wt* growth is inhibited. (**h**) *benA*^N100I^ and *GCN3*-1 have an additive effect on plocabulin resistance.

To test whether *A*. *nidulans GCN3*-1 is a loss-of-function mutation, we compared it to a *gcn3∆* mutant lacking the complete AN0167 ORF [yeast *GCN3* is not essential]^40,42^ (Fig. 6a). *A*. *nidulans gcn3*Δ grew like the *wt*, was not cryosensitive and did not display plocabulin resistance, unlike *GCN3*-1, suggesting that *GCN3*-1 is not a loss-of-function mutation (Fig. 2a,d). We next tested *GCN3*-1 and *gcn3*Δ sensitivity to 3-amino-1,2,4-triazol (3-AT), a competitive inhibitor of histidine biosynthesis that causes His starvation, inducing the GAAC pathway. Both the *wt* and *GCN3*-1 were resistant to 2.5 mM 3-AT, whereas *gcn3*Δ was hypersensitive (Fig. 6b), suggesting that at this 3-AT concentration the GAAC pathway was activated in a *gcn3*-dependent way and was necessary for survival in the *wt*. Doubling 3-AT concentration to 5 mM abolished *wt* but not *GCN3*-1 growth (Fig. 6b), strongly indicating that *GCN3*-1 is a gain-of-function allele resulting in hyper-activation of GAAC.

As the D^R^ mutant bore phenotypic similarities to C^R^ (*GCN3*-1), we also tested its response to 3-AT. D^R^ was resistant to 5 mM 3-AT as was *GCN3*-1 (Fig. 6b), suggesting that it also results in hyper-activation of GAAC. Prompted by this observation, we sequenced the genes for all regulatory subunits of eIF2B (Gcn3/AN0167/eIF2Bα, Gcd7/AN1344/eIF2Bβ, Gcd2/AN6864/eIF2Bδ) in the D^R^ strain and, indeed, detected a g. 1408 T > A substitution in AN1344, resulting in Tyr417Asn in the *A*. *nidulans* Gcd7 homologue. Genetic crosses showed that the mutant *gcd7* allele, *gcd7*-1, is linked to plocabulin resistance.

Since the D^R^ mutant strain displays 3-AT resistance, i.e. a Gcd^−^ phenotype, the causative Tyr417Asn substitution must lead to Gcd7 loss of function. In yeast, substitution in Gcd7/eIF2Bβ of the equivalent Phe360 (see Supplementary Fig. S4 alignment of *A*. *nidulans*, *S*. *cerevisiae*, *Homo sapiens* and *Schizosaccharomyces pombe* eIF2B α and β) and of the neighboring amino acid residues Pro358 and Ser359 by Ala is lethal^43^ and reduces co-immunoprecipitation of eIF2 with eIF2B, suggesting that a critical function of Gcd7 is stabilizing the eIF2::eIF2B interaction^43^. Indeed, the atomic structure of *S*. *pombe* eIF2B complex showed that Gcd7 Tyr377 (equivalent to Tyr41λn *A*. *nidulans* and Phe360 in *S*. *cerevisiae*) is located in the interface between regulatory subunits, within the central cavity that mediates binding to eIF2/eIF2α-P^35^ (Fig. 6c).

The observation that Gcd^−^ mutations in different eIF2B subunits conferred plocabulin resistance suggested that resistance was mediated by the upregulation of the GAAC/CPC pathway. We asked whether *GCN3*-1 and *gcd7*-1 would have a synergistic effect. However, from the cross between *GCN3*-1 and *gcd7*-1 we recovered three phenotypic classes in 1:1:1 ratio, corresponding to *wt* and to the single *GCN3*-1 and *gcd7*-1 mutants, which indicated that ascospores of the double mutant were unviable (see Materials and Methods for details). In yeast, the combination of constitutively de-repressing *GCN3* and *gcd2* alleles also leads to negative synthetic effects^44^. Synthetic lethality would be consistent with both *GCN3*-1 and *gcd7*-1 diminishing eIF2B GEF function by affecting independent molecular interactions within the eIF2B::eIF2 supercomplex.

We next asked whether *gcn3*Δ would modify *gcd7*-1 resistance to plocabulin. *gcn3*Δ improved growth of *gcd7*-1 at 37 °C and suppressed its cryosensitivity (Figs 2a and 6d). The double mutant was instead thermosensitive at 42 °C (Fig. 6d) and, unlike the single mutants, showed normal sensitivity to 3-AT (Fig. 6d). 3-AT normosensitivity may be explained as an intermediate phenotype between hypersensitivity of *gcn3*Δ and hyper-resistance of *gcd7*-1. Therefore, by reducing or eliminating the GAAC stress response, *gcn3*Δ may counteract GAAC de-repression mediated by *gcd7*-1. At the same time, general translation levels would increase in the double mutant compared to the single *gcd7*-1 mutant, which may explain growth restoration at both optimal and lower temperatures. Synthetic thermosensitivity, on the other hand, may result from increased instability of the double mutant eIF2B. Interestingly, resistance to plocabulin is only weakly decreased in the double *gcn3*Δ *gcd7*-1 mutant with respect to single *gcd7*-1 (Figs 2a and 6d), indicating that 3-AT normosensitivity is not diagnostic for plocabulin sensitivity.

We next tested whether components of the GAAC pathway acting downstream of eIF2B are required for plocabulin resistance in eIF2B mutants. We first deleted *gcn4/cpcA*^45^. *gcn4*Δ and *wt* strains were inoculated on medium containing 2.5 (Fig. 6e), 5 or 10 µM (Fig. 2a) plocabulin and incubated until the *wt* (whose growth was strongly inhibited) formed a microcolony. *gcn4*Δ showed hypersensitivity, which was very clear in the 2.5 µM sub-lethal plocabulin concentration (Fig. 6e), suggesting that Gcn4 counteracts plocabulin toxicity. To test whether *GCN3*-1 resistance also requires Gcn4, we set a genetic cross between *GCN3*-1 and *gcn4*Δ strains. However, we did not recover double mutant progeny, despite that *GCN3*-1 and *gcn4*Δ parental mutant and recombinant *wt* progeny appeared at the expected ratios, indicating that the double mutant progeny is unviable. Gcn4-dependent gene expression would then be essential for survival when general translation is reduced by *GCN3*-1.

We next ablated Gcn5/GcnE, a SAGA complex histone acetyltransferase^46^, necessary in *S*. *cerevisiae* for normal transcriptional activation by Gcn4^47^. *A*. *nidulans* SAGA complex mediates induction of some gene clusters but represses others^48,49^. GcnE is dispensable for growth, but necessary for conidiation^49,50^. Indeed, deletion of the entire *gcnE* ORF greatly impaired but did not abolish hyphal growth, whereas it abolished conidiation (Fig. 6f). *gcnE*Δ was slightly hypersensitive to 5 µM plocabulin (Fig. 6f), possibly reflecting the mutant’s growth defect. By meiotic crossing, we obtained *GCN3*-1 *gcnE*Δ double mutants, which grew similarly to *gcnE*Δ (Fig. 6f) and were additionally cryosensitive, like *GCN3*-1 (not shown). Although to a lesser extent than *GCN3*-1 single mutant, the double mutant was still resistant to plocabulin (Fig. 6f), indicating that resistance in *GCN3*-1 is largely GcnE independent. Unexpectedly, *gcnE*Δ (and *GCN3*-1 *gcnE*Δ) mutants were resistant to 5 mM 3-AT (Fig. 6g), indicating diverged roles of *A*. *nidulans* GcnE and yeast Gcn5.

Since interphase MTs of the C^R^ mutant displayed the strongest resistance to plocabulin (Fig. 4), we asked whether *benA*^N100I^ and *GCN3*-1 would have a synthetic effect. Notably, *benA*^N100I^ and *GCN3*-1 showed a strong additive effect on plocabulin resistance, the double mutant being almost insensitive to the drug (Figs 2a and 6h). As the residual plocabulin sensitivity of *benA*^*N100I*^ depends on TubC (see above), it is possible that *GCN3*-1 modifies the role that TubC plays in the susceptibility to plocabulin by counteracting, directly or indirectly, MT depolymerization or by altering the TubC/BenA expression profile.

### Substitution of a TIM44 amino acid residue critical for mitochondrial protein import reduces growth-inhibitory effect of plocabulin

The E^R^ mutation conferred moderate-to-low resistance to 5–50 µM plocabulin at 37 °C (Fig. 2a). E^R^ resistance was cryosensitive, whereas E^R^ growth was slightly thermosensitive and the E^R^ strain was defective in conidiation (Fig. 2a). These phenotypes were linked to resistance (no recombinants were obtained out of 48 progeny).

To identify the causative mutation, we assigned E^R^ to chromosome VIII by haploidization analysis. We then identified nucleotide polymorphisms on chromosome VIII by WGS. ≈250 polymorphisms were rejected because they also appeared in the parental strain used for mutagenesis or in the A^R^ and F^R^ mutants (A^R^ and F^R^ map to different linkage groups). Five candidate polymorphisms were E^R^-specific (indicated with lower case roman numerals in Supplementary Table S4).

**Figure 7:**
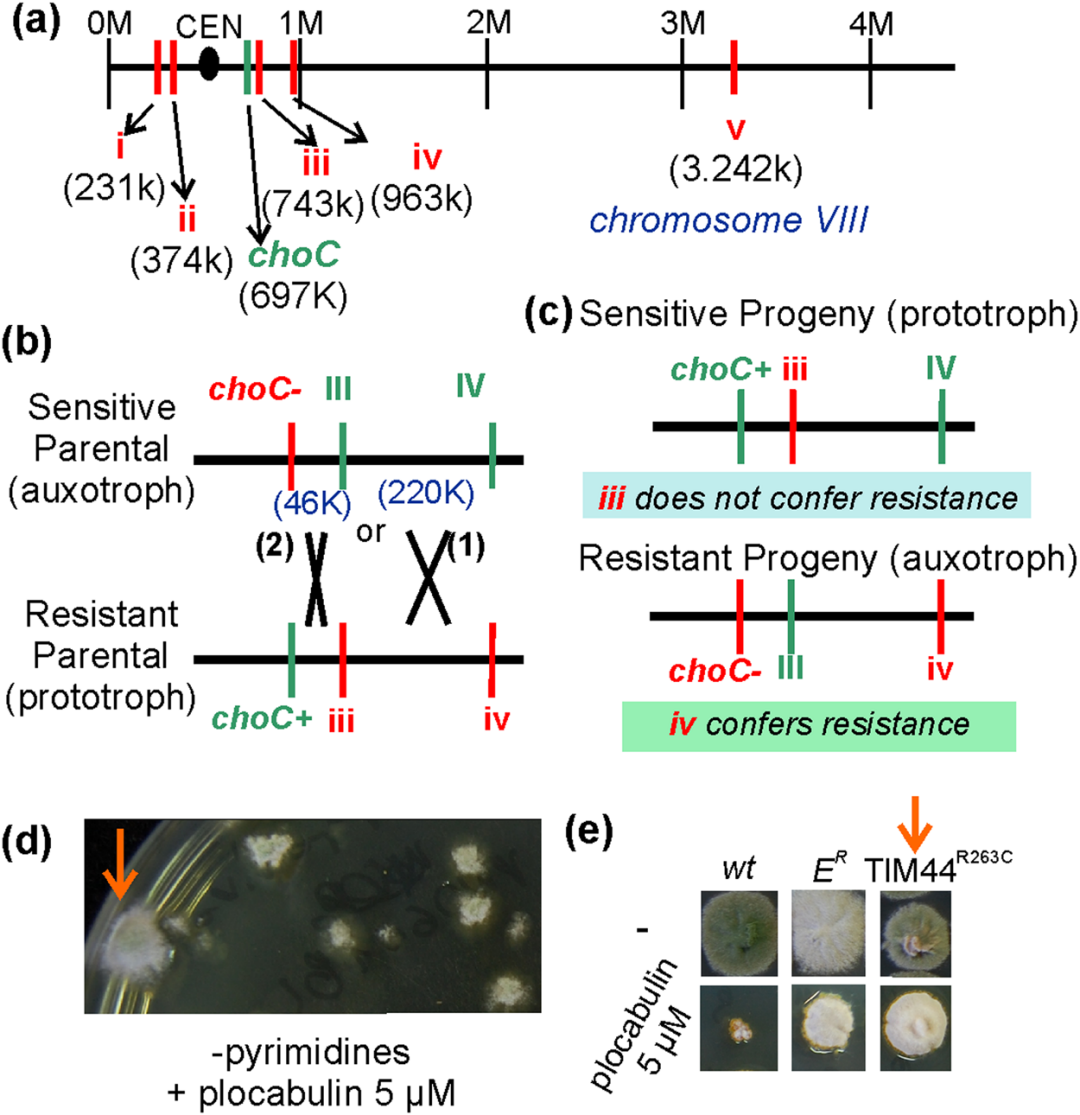
E^R^ plocabulin resistance is due to TIM44^R263C^ affecting mitochondrial protein import. (**a**) Position on chromosome VIII of polymorphisms i through v identified in the E^R^ genome. iii and iv are linked to *choC*, as is plocabulin resistance. (**b**) iii and iv are closely linked and both co-segregated with resistance. A three-point-cross between a choline prototroph (*choC* + ) E^R^ and a choline auxotroph (*choC*-) *wt* (E^S^, plocabulin sensitive) was used to determine the resistance-causative polymorphism. We selected progeny with recombined characteristics (choline auxotrophs/plocabulin resistant or choline prototrophs/plocabulin sensitive), i.e. recombination events within the *choC*/IV 260 kbp region. As distance between III and IV (220 kbp) is much larger than between *choC* and III, recombinants originating from a crossover in the III to IV interval (crossover 1) are more frequent, but they would only appear if they satisfied the selection criteria, i.e. if polymorphism iv were conferring resistance. If instead polymorphism iii were required for plocabulin resistance, then selected progeny would originate from a crossover within the *choC*/III region (crossover 2). (**c**) Three out of three choline prototroph, plocabulin sensitive progeny had originated from crossover (1), showing that polymorphism iii is not sufficient for resistance. A choline auxotroph, plocabulin resistant strain had originated from crossover (1), showing that polymorphism iv suffices to confer resistance. (**d**) By co-transformation of a pyrimidine prototroph strain with *pyrG* and a DNA fragment carrying polymorphism iv and by selection for pyrimidine prototrophy on plocabulin, we obtained 21 colonies, of which one (arrow, upper panel) showed plocabulin resistance after purification (arrow, lower panel). This transformant displayed an E^R^-like growth defect and had incorporated polymorphism iv into AN1281/*tim44*.

Meiotic crossing detected linkage of E^R^ to *choC*. Among the E^R^-specific polymorphisms, two (iii and iv, Fig. 7a) were physically linked to *choC*. We pooled PCR-generated DNA fragments of these two polymorphic loci from nine E^R^ (resistant) progeny of an outcross between an E^R^ strain (iii, iv) and the *wt* (III, IV) and sequenced them. Only the mutant nucleotide peaks were present in the sequencing chromatographs, indicating co-segregation of polymorphisms iii and iv with resistance, and implying that one (or potentially both) of them was causative. We next exploited linkage between loci iii and iv and landmarks in the region (*choC*-46 kb-III-220 kb-IV) to identify the causative mutation. We performed a three-point-cross between *choC* + iii iv and *choC*- III IV parental strains and selected for progeny with recombinant plocabulin resistance and choline auxotrophy traits (i.e. choline-independent, E^S^ and choline-dependent, E^R^ progeny) (Fig. 7b). Should the causative mutation have been iv, progeny arising from a single cross-over between *choC* and the E^R^-specific mutation that were plocabulin sensitive would have had a higher probability of carrying iii and IV (crossover 1, Fig. 7b) than III and IV (crossover 2). On the contrary, should the causative mutation have been iii, the only way to get plocabulin sensitive strains would have been the rare crossover 2 between *choC* and iii, leading to progeny carrying III and IV. Sequencing of three recombinant plocabulin sensitive progeny demonstrated that they carry iii and IV (i.e. it had arisen from crossover 1) (Fig. 7c), showing that polymorphism iii does not confer plocabulin resistance and indicating that polymorphism iv is instead causative. Sequencing of one plocabulin-resistant recombinant showed that it carried III and iv (i.e. the reciprocal progeny of crossover 1) (Fig. 7c), indicating that polymorphism iv alone is sufficient to confer E^R^ resistance to plocabulin.

Polymorphism iv is a missense mutation in AN1281. We reconstructed it by genetic engineering in a *wt* background, using a mutant linear DNA AN1281 fragment containing iv to replace the *wt* genomic DNA, co-selecting for a transformation marker (*pyrG*) and plocabulin resistance (Fig. 7d). We were able to recover one pyrimidine prototroph transformant that was plocabulin-resistant, which we showed to carry the correct gene replacement. This strain displayed all E^R^ characteristic phenotypes: plocabulin resistance, thermosensitivity and deficient conidiation (Fig. 7e), thus establishing that polymorphism iv confers resistance.

AN1281 codes for a conserved and essential component of the *P*resequence translocase-*A*ssociated *M*otor (PAM) complex, Tim44. PAM is recruited to the inner mitochondrial membrane translocation channel, TIM23, to complete translocation of mitochondrial matrix proteins^51^. Tim44 binds the imported protein precursor as it emerges from the TIM23 channel and recruits Hsp70 (yeast Ssc1)-ATP. Hsp70-mediated ATP hydrolysis, stimulated by PAM18, leads to tight binding of Hsp70 to the precursor protein and to subsequent release of the Hsp70::precursor protein complex from Tim44 into the matrix. Another molecule of Hsp70-ATP can then associate with Tim44 at the translocon exit to initiate a new cycle of ATP hydrolysis >precursor binding >matrix release, such that the precursor protein is being transported in a rachet-like fashion [reviewed in]^51^. Tim44 has an N-terminal domain contacting Tim23, Hsp70 and PAM16-PAM18 and a C-terminal domain contacting Tim17 and the translocating precursor proteins; it has been proposed that rearrangements of the two domains, possibly triggered by incoming protein traffic, drive protein translocation^52^.

The E^R^ mutation leads to the Arg263Cys substitution within the N-terminal domain of AN1281/Tim44 (Supplementary Fig. S5). The equivalent amino acid residue in *S*. *cerevisiae* Tim44, Arg180, is critical for the polypeptide substrate-induced destabilization of the Ssc1(Hsp70)-Tim44 complex and for the association of Tim44 with the TIM23 translocase^53^. Arg180Ala-Tim44 in yeast results in growth defects^53^, in line with the conidiation and growth defect displayed by Arg263Cys-Tim44 *A*. *nidulans*. Given the conservation of Tim44 homologues, Arg263Cys may have similar effects on *A*. *nidulans* Tim44 to those caused by Arg180Ala on the *S*. *cerevisiae* protein, suggesting that an altered mitochondrial protein import would confer resistance to plocabulin.

### Substitutions in the middle homology region of two binuclear zinc cluster transcription factors confer resistance to plocabulin

The A^R^ mutant conferred the highest resistance to 5–50 µM plocabulin at 37 °C among the mutants obtained from the genetic screen (Fig. 2a). A^R^ showed a slight conidiation defect at 37 °C, exacerbated at 25 °C. A^R^ resistance to plocabulin was partially thermosensitive (Fig. 2a).

Using WGS and comparing the mutant genome with that of the strain used for mutagenesis, we identified 21 polymorphisms in A^R^, potentially resulting in missense or truncating mutations. By haploidization analysis, we assigned the plocabulin resistance mutation to chromosome II, which contained three of these mutations, affecting AN3969, AN4084 and AN8292 (Supplementary Table S5).

**Figure 8:**
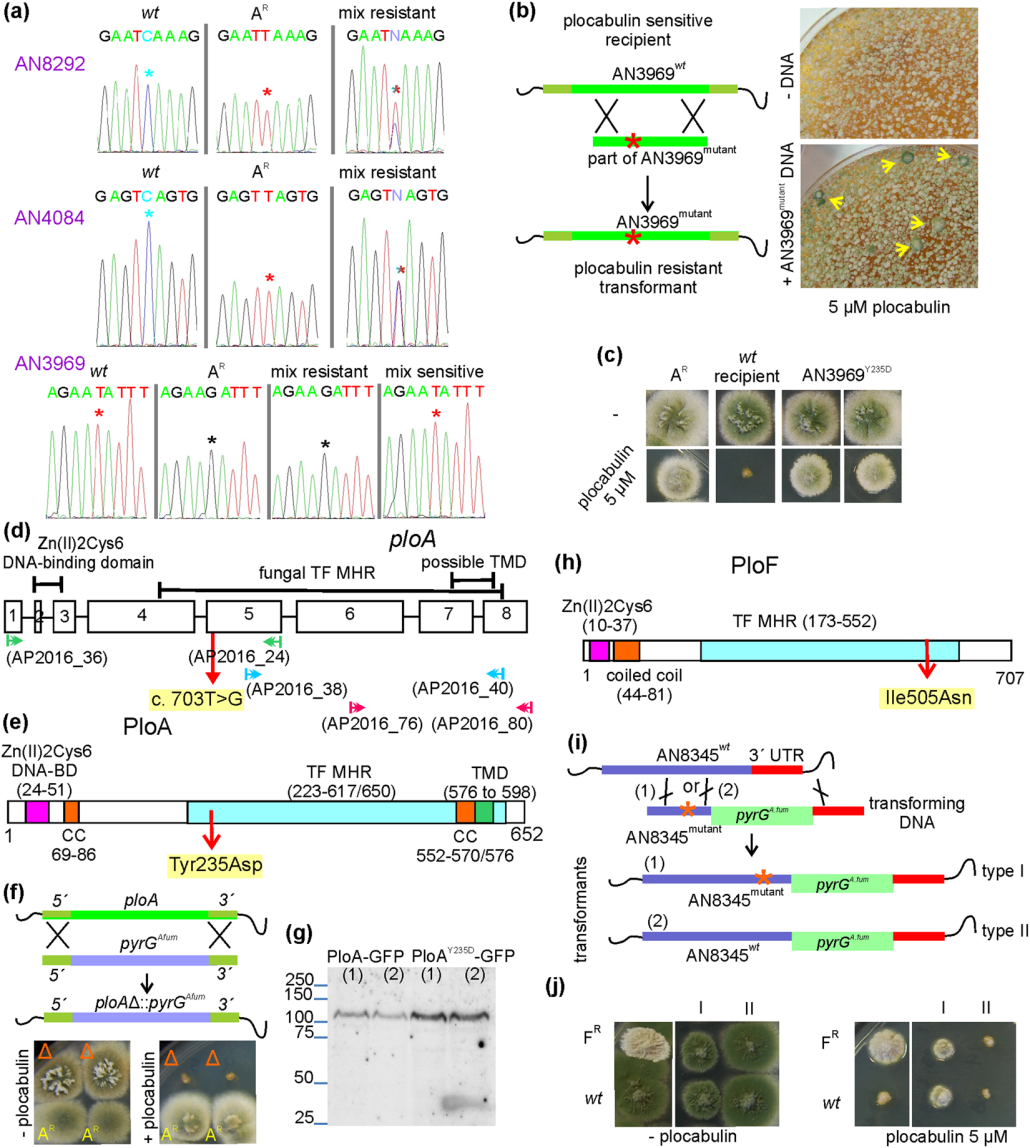
A^R^ and F^R^ resistance is conferred by mutations in the middle homology region of Zn2Cys6 binuclear zinc cluster transcription factors. (**a**) Sequencing chromatographs of PCR-amplified fragments of the indicated genes from a *wt* and an A^R^ strain and from pools of 12 plocabulin resistant or sensitive progeny from a cross between the *wt* and the A^R^. Note, in the mix from resistant strains, the double peaks for genes AN8292 and AN4084 in the position (indicated by an asterisk) of polymorphisms found in the A^R^ strain (MAD4738) by WGS, indicating that these polymorphisms do not co-segregate with resistance. Note the single peak observed instead for polymorphism in AN3969 from resistant or sensitive progeny pools, with the nucleotide in the resistant population corresponding to the polymorphism found in the A^R^ while that from the sensitive population to the *wt* sequence. (**b**) Strategy to reconstruct polymorphism AN3969 in a *wt* recipient strain (left) and direct selection for plocabulin-resistance after transformation (right). Colonies that have incorporated the mutation are large and produce green conidia (yellow arrows) on plocabulin. (**c**) Transformants were indistinguishable from the original A^R^ strain, confirming that AN3969 polymorhism confers the A^R^ phenotype. (**d**) AN3969 gene model based on amplification and sequencing of cDNA with the DNA primers indicated in the scheme (S2 Table). The start codon is uncertain. Exon coordinates on chromosome II: (exon 1) 2.476.638-2.476.570, (2) 2.476.510-2.476.487, (3) 2.476.423-2.476.323, (4) 2.476.259-2.475.754, (5) 2.475.693-2.475.359, (6) 2.475.261-2.474.800, (7) 2.474.719-2.474.478, (8) 2.474.423-2.474.204. (**e**) AN3969/PloA architecture: Tyr235Asp confers plocabulin resistance. The zinc binuclear cluster domain [Zn(II)2Cys6], followed by a coiled-coil domain, as well as the MHR regulatory region, are characteristics of this family of transcription factors. (**f**) Strategy for deletion of the AN3969/*ploA* ORF and sensitivity to plocabulin displayed by AN3969Δ (Δ). (**g**) Anti-GFP western blot of C-terminally tagged AN3969^wt^ and AN3969^Y235D^ with GFP. (**h**) AN8345/PloF domain organization. (**i**) Strategy for reconstructing AN8345 polymorphism by *in locus* integration of a DNA fragment with the mutation, followed by *pyrG*^Af^. Double homologous recombination results in pyrimidine prototrophy when a crossover in the 3′UTR region is combined with either crossover 1 (incorporating the polymorphism in the genome) or crossover 2 (without polymorphism incorporation). (**j**) Two types of transformants were obtained by transformation with the PloF DNA fragment: Type I had incorporated the mutation, grew compact and displayed plocabulin resistance, although less than the original F^R^ mutant. Type II that had not incorporated the polymorphism, grew as the *wt* and was sensitive to plocabulin. This showed that Ile505Asn in AN8345 confers plocabulin resistance.

We crossed the A^R^ with a *wt* strain and checked if any of the candidate mutations co-segregated with plocabulin resistance. To streamline this analysis, we PCR-amplified each of the three candidate genetic loci from 12 plocabulin-resistant progeny, mixed equal quantities of the 12 individual PCR products for each locus and sequenced the resulting mixtures. Overlapping *wt* and mutant nucleotide peaks were identified in the positions of the AN4084 and AN8292 polymorphisms in the corresponding sequencing chromatograms (Fig. 8a). On the contrary, a single peak was observed at the position of the AN3969 polymorphism and this peak corresponded to the nucleotide identified in the A^R^ mutant (Fig. 8a). To buttress this result, we then sequenced a pool of AN3969 DNAs from 12 plocabulin-sensitive progeny. A single peak in the chromatogram (Fig. 8a) indicated that sensitive strains were also homogeneous with regard to that nucleotide position, and showed that the identified nucleotide in this case corresponded to the *wt* AN3969 allele. These results strongly indicated the AN3969 polymorphism was causative of the A^R^ plocabulin resistance.

To demonstrate this contention, we re-introduced the AN3969 mutation into a *wt* strain by transformation, directly selecting transformants on plocabulin. Large conidiating colonies could be singled out over the background of non-transformed cells (Fig. 8b). After purifying these colonies, we verified that they had incorporated the relevant AN3969 mutation, and confirmed that their growth with or without the drug was indistinguishable from that of the original A^R^ strain (Fig. 8c). This showed that AN3969 polymorphism confers the A^R^ plocabulin resistance. We denoted AN3969, thus far uncharacterized in fungi, *ploA* (*plo*cabulin resistant class *A*)

PloA is predicted to include a fungal transcription factor (TF) regulatory middle homology region (MHR), but, according to the genomic database (www.aspgd.org), it would lack the N-terminal binuclear zinc cluster DNA-binding domain normally associated with the MHR^54^. By PCR amplification from a total cDNA pool, we found that AN3969 encodes an mRNA much larger than predicted. Figure 8d shows a corrected gene structure for *ploA*, as deduced from cDNA sequencing. Once corrected, *ploA* includes coding information for a canonical fungal-specific GAL4-like Zn(II)2Cys6 binuclear cluster DNA-binding domain^55^. PloA is predicted to have coiled-coil domains in addition to at least one transmembrane domain towards its C-terminus (Fig. 8e). The Zn(II)2Cys6 binuclear cluster protein family is composed of fungal-specific TFs playing multiple regulatory roles including in multi-drug resistance (yeast PDR1/3), sterol sensing and regulation of sterol biosynthetic genes (ECM22/UPC2), mitosis (kinetochore protein CEP3) and chromatin remodeling^56^. Their activity is modulated by small molecules such as chemicals, nutrients and metabolites^57^. According to the most probable PloA N-terminus based on cDNA sequencing (Fig. 8d), class A mutation c. 703 T > G results in Tyr235Asp substitution within the putative regulatory PloA MHR.

We deleted *ploA* to test whether loss of function would confer plocabulin resistance. *ploA∆* grew normally and did not show resistance to plocabulin (Fig. 8f), suggesting that Tyr235Asp leads to gain- or modification-of-function. This conclusion is in line with previous reports indicating that the MHR domain plays an inhibitory role in the transcriptional activity of zinc cluster proteins^56^.

To gain insight into the function of PloA we investigated its localization after GFP tagging. If the protein fusion were functional, GFP-tagged Tyr235Asp-PloA should confer plocabulin resistance. Instead N-terminal GFP tagging abolished resistance. C-terminally tagged Tyr223Asp-AN3969 permitted partial plocabulin resistance (not shown). Anti-GFP western blotting revealed the presence of a faint band showing a slightly slower mobility than the expected from the fusion protein size (Fig. 8g). Hardly any fluorescence was detectable with the *wt* PloA-GFP protein, while a minority of Tyr223Asp-PloA-GFP hyphae showed clustered foci of fluorescence with a subcellular distribution that would be consistent with nuclear association (not shown).

A second zinc cluster family protein appeared to be responsible for the moderately resistant F^R^ mutant. Plocabulin resistance in F^R^ was partially thermosensitive (Fig. 2a). Chromosomal allocation localized the F^R^ mutation on chromosome V, where three potentially causative polymorphisms in genes AN8345, AN12394 and AN5786 were identified by WGS (Supplementary Table S6). A genetic cross between the F^R^ and a *wt* strain, followed by sequencing of candidate *loci* in plocabulin resistant and sensitive progeny showed that polymorphism AN8345 co-segregated with resistance, unlike polymorphisms in AN12394 and AN5786, suggesting that a mutant AN8345 conferred F^R^ resistance.

According to the *A*. *nidulans* genome sequence annotation, the F^R^ AN8345 polymorphism g. 1885 T > A lies in the middle of an abnormally large, 138 bp-long seventh intron. Inspection of RNAseq data unambiguously showed that the intron acceptor site lies upstream from the predicted site, producing a more typical 66 bp intron. Corrected accordingly, AN8345 product has 24 additional amino acid residues, encoded by the beginning of exon 8, which would be part of the AN8345 MHR domain. In this corrected gene model, the F^R^ polymorphism would fall in exon 8, resulting in substitution Ile505Asn within the MHR (Fig. 8h).

We were unable to reconstruct the F^R^ mutation by transformation and direct selection on plocabulin or by co-transformation with *pyrG* DNA and selection for pyrimidine prototrophy, like in the cases of PloA/A^R^, Tim44/E^R^ and Gcn3/C^R^. Therefore, we constructed a linear DNA molecule consisting of a C-terminal AN8345 fragment containing the F^R^ mutation, *A*. *fumigatus pyrG* and AN8345 3′UTR. The presence of *pyrG*^*Af*^ permits selection of the desired gene replacement by a double crossover in the AN8345 locus (Fig. 8i). Depending on the position of the N-proximal crossover the F^R^ mutation is incorporated in a proportion of gene replacement events. We obtained two types of transformants with regard to colony growth: *wt*, which were sensitive to plocabulin, and compact, which showed substantial plocabulin resistance, albeit weaker that the original F^R^ mutant (Fig. 8j). We verified by sequencing that plocabulin-resistant strains had incorporated the F^R^ mutation, while plocabulin-sensitive strains had instead the *wt* AN8345 allele. Both lower levels of resistance and compact growth may result from insertion of *pyrG* immediately downstream the AN8345 ORF, which removes the ‘natural’ 3′UTR from AN8345 transcript and may interfere with the promoter of the next gene, AN12339. Thus, with the caveat that we could only partially recapitulate the resistance displayed by F^R^, the above experiments strongly indicate that AN8345 polymorphism is causative of the F^R^ plocabulin resistance.

We denoted the uncharacterized AN8345 gene as *ploF*. *ploF* codes for a protein containing a Zn(II)Cys6 binuclear cluster and the associated MHR (Fig. 8h). Interestingly, the divergently transcribed gene AN8344, located upstream from AN8345 (≈ 400 bp separate the two start codons) codes for a homologue of PDR15, a plasma membrane multidrug transporter of the ATP binding cassette (ABC)-family that is regulated by zinc cluster TFs in response to stress^58^. We observed a similar arrangement of an ABC transporter and a zinc cluster protein in AN8345 homologues from *Aspergillus oryzae* and *Aspergillus niger*, suggesting that the two genes are under common regulation, which would implicate the F^R^ Zn(II)Cys6 binuclear cluster TF in multidrug resistance responses including the expression of multidrug efflux pumps.

## Discussion

Fungi are a sister taxonomic group to metazoa. Therefore, many antitumor compounds have concomitant antifungal activity, which is also the case for plocabulin. Given the importance of MTs for filamentous fungal cells, and the many precedents in MT discovery in filamentous fungi, we reasoned that mutants of *A*. *nidulans* resistant to plocabulin would not only provide information relevant to its MoA, but they would also help define cellular pathways by which tumor cells might be able to overcome the anti-proliferative activity of plocabulin. Using the *Aspergillus* genetic toolbox, we established causative relationship between mutations in several genes and resistance to plocabulin.

A major achievement was the generation of a completely resistant mutant by combining a binding site-disrupting mutation in the major β-tubulin with deletion of the minor β-tubulin gene, such that all cellular β-tubulin was of the “resistant-type”, BenA^N100I^. This positive result showed that, up to the maximum concentration used, plocabulin has a unique molecular target relevant to fungal growth, β-tubulin, and underlined the fact that in fungi the available tubulin isotypes co-determine the response of microtubules to the drug. Knowing the spectrum of their molecular targets is crucial to comprehend and regulate the drug effects at the cellular and organismal level. One case in support of this statement is the shift in the perception of the MoA of anti-MT compounds^59,60^. Although it has long been thought that anti-MT compounds kill cells by blocking mitosis, human tumors divide rather slowly and the primary toxicity associated with the administration of anti-MT agents, neurotoxicity, concerns non-dividing cells^60^. This is considered as evidence that anti-MT compounds induce cell death independently of mitosis; it has been proposed that they attack interphase microtubules instead^60^. However the need to confidently rule out the existence of secondary drug targets becomes most apparent. Ruling out secondary targets was important to assess the mechanistic basis of resistance conferred by the non-tubulin *A*. *nidulans* mutations: if only one plocabulin target is assumed, these mutations must affect drug availability, microtubule dynamics or the consequences of depolymerizing microtubules.

Another mutant that was nearly insensitive to the drug, although at the expense of debilitated growth, was the double *benA*^N100I^ *GCN3*-1 mutant. *benA*^N100I^ *GCN3*-1 resistance to plocabulin suggested that *GCN3*-1 diminishes the sensitizing effect that the minor β-tubulin isotype, TubC, contributes to the response to plocabulin. *GCN3*-1 is the mutant with the most plocabulin-resistant interphase MTs, despite that *GCN3*-1 colony formation on plocabulin was more debilitated than in other mutants. That drug-resistant interphase MTs do not directly translate into resistant growth suggests that MT resistance in *GCN3*-1 cannot be solely attributed to a reduction in intracellular drug concentration, as this would restore growth proportionately. Instead, *GCN3*-1 may have an effect directly on MTs. A plausible explanation would be that *GCN3*-1 triggers an increase in BenA expression that would predictably increase the drug concentration threshold needed to overrun tubulin and that may cause growth defects related to abnormal MT stability. Such hypothetical increase in BenA expression, if combined with an inefficient plocabulin binding site on BenA, as we demonstrate to be the case for *benA*^N100I^, might give rise to complete plocabulin resistance.

The finding that mutations in subunits α and β of eIF2B that display a Gcd^−^ phenotype (constitutive GAAC activation) confer plocabulin resistance is noteworthy. Constitutive mutations in *GCN3* have been previously isolated in *S*. *cerevisiae*^44^ and most of them appear to interfere with interactions between the N- and the C-terminal domains of eIF2Bα^61^. Ser149Phe substitution in *GCN3*-1 is a novel gain of function mutation in eIF2Bα. The conserved human eIF2Bα-Ser131 is positioned at the center of a positively charged inter-domain pocket^61^. eIF2Bβ Tyr417 that confers plocabulin resistance when substituted by Asn in *gcd7*-1 appears to be located at the interface of eIF2B subunits β and δ, within the central cavity accommodating eIF2α^35^. Its interaction with Asn460 of subunit δ might be important for maintaining the bedrock of this central cavity (see Fig. 6c). The isolation of GAAC pathway-activating mutations in two different eIF2B subunits strongly argues for a causative role of GAAC activation and/or general translation reduction in plocabulin resistance. However, the double *gcd7*-1 *gcn3*Δ mutant nears the plocabulin resistance of *gcd7*-1, although it presents normal sensitivity to 3-AT, unlike its constituents, *gcd7*-1 (hyper-resistant) and *gcn3*Δ (hyper-sensitive). Would this indicate that GAAC activation and plocabulin resistance are separable? It is likely that, despite the apparent normosensitivity to 3-AT, the GAAC would still be constitutively activated in the double mutant background, possibly because eIF2α would not be optimally accommodated by eIF2B due to the presence of Gcd7(Tyr417Asn). Loss of Gcn3 from the Gcd7(Tyr417Asn)-containing eIF2B may however lead to lower constitutive activation, which would explain the remediation in the double mutant of the growth defect and cryosensitivity displayed by the single *gcd7*-1 mutant. Upon stimulus, there would be no eIF2α-P-mediated induction of the GAAC in the *gcd7*-1 *gcn3*Δ mutant, perhaps because enhanced interactions between the regulatory eIF2B subunit and eIF2α-P, thought to prevent catalytically productive eIF2B/eIF2 interaction^33^, depend on Gcn3. Thus, although the apparent response to starvation of the double mutant resembles the *wt* response, resistance to 2.5 mM 3-AT would be independent of eIF2α phosphorylation, unlike in the *wt*. It is plausible that this low, but constitutive GAAC activation seen in the double *gcn3*Δ *gcd7*-1 mutant causes plocabulin resistance under non-starvation conditions. Whether other GAAC-activating eIF2B mutations would confer plocabulin resistance merits further examination. It is worth noting that features of the solid tumor microenvironment such as hypoxia and nutrient deprivation have been associated with ATF4 overexpression regulated by the PERK/GCN2-eIF2α pathways and eIF2α phosphorylation has been shown to promote tumor growth^62,63^. It would thus appear that the ISR pathway may be constitutively activated in some tumors and it would be important to understand if and when this activation may be relevant for the response in therapeutic treatments with anti-MT agents.

We also identified Arg263Cys-Tim44 as causative of plocabulin resistance. Judging by the effect of analogous mutations in yeast homologues, this substitution most likely affects a critical function of TIM44 in pre-protein translocation into the mitochondrial matrix. In agreement, we found that TIM44^R263C^ expression in a strain co-expressing GFP-tagged Tom20 (an outer mitochondrial membrane translocase component) leads to a strong synthetic negative effect on growth (not shown). Identifying that mitochondrial function may affect plocabulin resistance could be important, as the efficiency of anti-MT agents has been linked to the apoptotic mitochondrial pathway^59,64^. The MT-mitochondria relationship appears also important for chemotherapy-induced peripheral neuropathy, as atypical mitochondria have been suspected to underlie neuron dysfunctions after treatment with MT targeting agents^64^.

Some of the non-tubulin mutations resulting in plocabulin resistance confer similar cross-resistance levels to benomyl, another anti-MT agent, suggesting they might target cellular “escape doors” downstream from the imposed MT depolymerization problem. These mutations would be particularly relevant in therapeutic treatments because they might provide resistance to a broad range of anti-microtubule agents. In fact, it is plausible that non-tubulin resistance mutations would be the primary therapeutic concern, as the presence of several β-tubulin isotypes in human cells would minimize the effect of mutations altering the drug-binding site of one particular β-tubulin isotype.

Finally, the finding that two TFs of the binuclear zinc cluster family are involved in plocabulin resistance was unsurprising, given the importance of these factors in multidrug resistance. The mutations obtained fall within the MHR region, whose deletion/mutation renders this type of TFs constitutively active^56^. Of note, at least the AN3969/*ploA*-associated mutation leads to gain of function. Interestingly, the two affected TFs display different cross-resistance to benomyl, suggesting that the pathways implicated are not overlapping. PloA is predicted to have one TMD towards its C-terminus, similar to *Candida albicans* Upc2, a binuclear cluster zinc family TF regulating ergosterol biosynthetic genes^56^. Microscopic studies monitoring the uptake of a dimethylaminocoumarin-plocabulin analogue by fluorescence microscopy suggested that Tyr223Asp in AN3969 might prevent drug uptake (not shown). It would be interesting to investigate whether this effect involves an alteration of plasma membrane lipid content. On the other hand, AN8345/*ploF*, whose chromosomal location suggests co-regulation with a PDR15 homologue, may act by regulating expression of drug efflux pumps.

In conclusion, our genetic analyses in *A*. *nidulans* have provided important insights relevant to the mechanism of action of plocabulin. They have also provided important resistance-associated hit proteins involved in metabolic processes that would potentially affect treatment outcomes. Given that, as in cell lines^65^, resistance hits involving mutations in the primary target were also identified by our screen, the presence of different tubulin isotypes was shown to be important also for fungal cells and fungal cell fitness has been shown to rest on both mitotic and interphase MTs, we have grounds to believe that it would be reasonable to pursue those novel hits to assess their importance in tumor environments.

## Materials and Methods

### *Aspergillus nidulans* growth media and techniques

Aspergillus standard complete (CM) or minimal medium (MM) (Cove 1966) was used for growth tests. Plocabulin and benomyl resistance were tested on CM, 3-aminotriazol resistance was tested on MM with 1% glucose, 10 mM ammonium tartrate and the appropriate vitamins. Transformations were as described^66^. Gene targeting strategies and analysis of transformants were as described in^31,67,68^.

### Microscopy

Microscopy was carried out with a Leica DMI6000B, as described^10^. MAD4659 (S1 Table) was crossed to obtain resistant progeny expressing *gfp::tubA* and *hhoA::mcherry*. For shift experiments plocabulin was added to pre-warmed fresh medium used to substitute the plocabulin-free medium in which hyphae had been growning overnight. GFP and mCherry channels were acquired successively by rapid change of the microscope filters driven by the multidimensional acquisition function of Metamorph (Molecular Devices).

### A genetic screen for plocabulin resistant mutants

We mutagenized conidiospores of strain MAD2 (S1 Table) by UV irradiation on plate using a UV stratalinker 1800. 10^6^ to 10^8^ viable spores per petri dish were plated on medium containing plocabulin 10 µM and left to grow in the dark. After 5 days, we picked colonies that had grown faster than a dense background of micro-colonies and streaked them on medium with plocabulin to clean from non-resistant cells. Colonies were further purified by plating spore dilutions on medium without plocabulin (to avoid further plocabulin-induced mutagenesis). Spore dilutions and plating were repeated until colonies appeared homogeneous. This gave rise to strains MAD4647 through MAD4653 (S1 Table). These were backcrossed with MAD3688 to test for Mendelian segregation of resistance and their *tubA* and *benA* genes were sequenced using DNA primers 1 to 8 (S2 Table) to test for the presence of mutations in the major tubulins.

### Chromosomal allocation

Diploid formation between MAD4629, a master strain carrying diagnostic markers in each chromosome, and MAD4744 (C^R^), MAD4738 (A^R^), MAD4747 (E^R^), MAD4751 (F^R^), as well as haploidization analysis were as previously described^30,69^.

### Whole genome sequencing

Genomic DNA for WGS was isolated using standard techniques. Genome sequencing and analysis of SNPs were carried out by Life Sequencing S.L. (www.lifesequencing.com) (Illumina HiSeq. 100PE, 50–100x, MAD4650) or Otogenetics corporation (www.otogenetics.com) [Illumina HiSeq. 2500, PE 100–125, 100x, MAD4738(A^R^), MAD4747 (E^R^), MAD4751 (F^R^) and MAD2 (*wt*)]. To pick candidate SNPs to cause resistance in each strain, we selected all missense, frameshift or 5′UTR mutations that were not present in the genomes of the strains with resistance mutations that were classified to different linkage groups. We visualized WGS data with the Integrative Genomics Viewer^70,71^.

### Construction of *tubC*Δ *benA*^*N100I*^

For *tubC* ORF deletion, an appropriate DNA fragment was constructed (fusion PCR, primers 41 to 46) (Fig. 1d) and used to transform MAD5319. *tubC*Δ (confirmed by Southern blot) strain MAD5755 was crossed with *benA*^*N100I*^ MAD4736 to acquire double mutants.

### C^R^ identification

To map the causative C^R^-resistance mutation, we crossed MAD4650, MAD4744, and MAD4745 (C^R^) with MAD4852, MAD4853, MAD4855, MAD873 and MAD1426 carrying appropriate markers on chromosome VIII. *GCN3*-1 was followed in genetic crosses by Sanger sequencing or XbaI digestion (*GCN3*-1 interrupts a XbaI site) of a DNA fragment amplified with primers 9 and 10 (S2 Table). We reconstructed *GCN3*-1 co-transforming MAD5319 with the *pyrG* of *A*. *nidulans* (amplified with primers 82 and 83) and a fragment of *gcn3* carrying *GCN3*-1 (amplified with primers 9 and 10 from MAD4744) and selecting for pyrimidine prototrophy and *GCN3*-1 growth/plocabulin resistance.

### D^R^ identification

Sequencing of eIF2B regulatory subunits genes *GCN3*/eIF2Bα (AN0167), *GCD7*/eIF2Bβ (AN1344) and *GCD2*/eIF2Bδ (AN6864) was carried out using primers 9, 10, and 19–23 (S2 Table).

### Construction of mutants in the GAAC pathway

#### *GCN3*-1 *benA*^*N100I*^

MAD4744 was crossed to MAD4757.

#### Construction of *gcn3*Δ

Primers 11–16 were used to construct the linear fragment *5*′*utr*^*gcn3*^::*pyrG*^*Af*^::*3*′*utr*^*gcn3*^ (Fig. 6a)^67^ that was used to transform MAD1739. *gcn3*Δ was verified by Southern blot analysis.

#### The double mutant *gcn3*-1 *gcd7*-1 is unviable

We crossed *GCN3*-1 (MAD5660) and *gcd7*-1 (MAD4740) and recovered three phenotypic classes (1:1:1 ratio) that were *wt-*, *GCN3*-1- and *gcd*7-1-like (see Supplementary Fig. S6 for details). We genotyped five *wt*–like, six *GCN3*-1-like and seven *gcd7*-1-like strains and confirmed that they carried one or the other or no mutation, but we did not recover any double mutant. Because *gcn3*/*gcd7* loci recombine freely (suggested by recovery of the *wt* strains at the expected 1/3 ratio), this indicated that the double *GCN*3-1 *gcd7*-1 mutant must be unviable.

#### *gcn3*Δ *gcd7*-1

We crossed MAD4740 (*gcd7*-1) with MAD5386 (*gcn3*Δ)

#### *gcn4*Δ

The appropriate construct for the deletion of *gcn4* (AN3675) (Fig. 6a) was constructed by fusion PCR using primers 68–73 and was used to transform MAD5319. Correct genome integration was verified by Southern blot analysis. *gcn4*Δ displayed compact growth. A genetic cross between a *pyrG*+; *gcn4*+ (MAD5660) and *pyrG89; gcn4*Δ::*pyrG*^*Af*^ (MAD5991) strain yielded *gcn4*Δ strains that were compact and *gcn4*Δ strains that were indistinguishable from *wt*. 3 compact *gcn4*Δ strains were *pyrG89*, while 3 non-compact *gcn4*Δ were pyrG+, strongly suggesting that compact growth was related to *pyrG89* (pyrimidine auxotrophy).This indicated that *pyrG*^*Af*^ only partially complements *pyrG89* when integrated in the *gcn4* locus.

#### *gcnE*Δ

The appropriate construct for the deletion of *gcnE* (AN3621) (Fig. 6a) was constructed using primers 74–79 and was used to transform MAD5319. Transformants were genotyped using primers 80 and 81. *gcnE*Δ *gcn3*-1 strains were obtained by crossing MAD6085 with MAD5660.

### A^R^ identification

To test which of the candidate mutations (S5 Table) co-segregated with resistance, MAD4738 (A^R^) was crossed to MAD3685 and, from the genome of progeny, DNA fragments were amplified that included the candidate polymorphisms’ regions. Primers 30 and 31 were used for AN4084 amplification, 32 and 33 for AN3969 and 34 and 36 for AN8292. PCR fragments from 12 plocabulin-resistant or 12 plocabulin-sensitive mutants were mixed and the mixtures sequenced. Two peaks in the chromatograph at the relevant polymorphism position were indicating that this polymorphism was not homogeneous in the population and, thus, it did not co-segregate with resistance, while single peaks from both the resistant and sensitive strains indicated that the polymorphism co-segregated with resistance (Fig. 8a).

#### A^R^ reconstruction

A DNA fragment with the AN3969 mutation was amplified from MAD4738 with primers 32 and 33 and was used to transform MAD5321. Transformants were selected on 5 µM plocabulin.

#### AN3969 cDNA amplification

From total *A*. *nidulans* cDNA, we managed to amplify overlapping AN3969 fragments with primers 33–36, 37–38 and 39–40 (Fig. 8a and S2 Table). Sequencing of these fragments verified the introns in the genome annotation, as well as the stop codon, but showed that a much larger transcript, extending towards the 5′prime, is expressed. This transcript contained a Zn2Cys6 binuclear cluster DNA binding motif.

#### PloA::GFP

A construct to tag AN3969/*ploA* with GFP at its C-terminus (*ploA*^*part*^::*gfp*::*pyrG*^*Af*^:: *3*′*utr*^*ploA*^) was constructed by fusion PCR using primers 39 and 57, 25 and 58 and 59 and 60 (*gfp*::*pyrG*^*Af*^ was amplified from p1439). The construct was used to transform MAD5736 (*wt*) and MAD6079 (A^R^), *pyrG89 nkuA*Δ*::bar* strains (i.e. appropriate for genome integration by homologous recombination of DNA and for selection of the transformants by pyrimidine auxotrophy) coming from the cross between MAD5914 and MAD5736. PCR genotyping of the transformants was with primers 39 and 61. To assess the size of the hybrid protein and its expression levels, total protein was extracted by the alkaline lysis method^72^. Samples were resolved in a 7.5% SDS-polyacrylamide gel and transferred to a nitrocellulose filter that was reacted with α-GFP antibody (1:5000; Roche). Peroxidase-coupled goat-anti mouse IgG (H + L) (1:5000; Jackson ImmunoResearch) was used as secondary antibody. Bands were detected using ECL (GE Pharmaceuticals, RPN2106).

#### AN3969Δ

To delete AN3969, primers 24 to 29 were used in fusion PCR (Fig. 8).

### E^R^ identification

Linkage assessment of E^R^ to *choC* and further analysis of the two linked polymorphisms iii and iv used progeny from a cross between MAD4747 (E^R^) and MAD5660 (*choC*^−^). Genes affected by iii and iv were amplified and sequenced using primers 47 and 48 (iv) or 49 and 50 (iii). We reconstructed E^R^ by transforming MAD5319 with *pyrG*^*Anid*^ and a fragment of AN1281 carrying the resistance mutation (amplified using primers 48 and 84) and selecting for pyrimidine prototrophy and plocabulin resistance.

### F^R^ identification

To test how candidate polymorphisms in F^R^ strains segregate relative to plocabulin resistance, we crossed MAD4751 (F^R^) to MAD3685. Polymorphisms were sequenced in PCR pools from resistant or sensitive progeny, using primers 51 and 52 (AN5786), 53 and 54 (AN8345), and 55 and 56 (AN12394). Reconstruction of AN8345/*ploF* polymorphism co-segregating with resistance was achieved by transformation of strain MAD5736 with the appropriate construct (Fig. 8i) carrying part of the mutant AN8345 ORF, followed by the *pyrG*^*Af*^ and 3′UTR of AN8345. Primers 62 to 67 were used to amplify and fuse the appropriate DNA fragments.

### Availability of materials and data

Strains, materials, data and protocols will be made available to readers upon well-founded and reasonable request. Requests for Plocabulin must be addressed to Pharmamar SA, a for-profit company, and can be obtained only through a Material Transfer Agreement with Pharmamar, that will implement a detailed analysis of the scientific proposal.

## Electronic supplementary material

Supplementary information

S1 Movie

S2 Movie
