# Supplementary material for: Molecular basis of resistance to the microtubule-depolymerizing antitumor compound plocabulin
Source: Sci Rep. 2018 Jun 5;8:8616. doi: 10.1038/s41598-018-26736-3 (PMC5988728; doi:10.1038/s41598-018-26736-3)
Supplement: Supplementary file 1 — Supplementary information [file 41598_2018_26736_MOESM1_ESM.pdf]

Supplementary information for the article:

Molecular basis of resistance to the microtubule-depolymerizing antitumor compound plocabulin

by Areti Pantazopoulou, Carlos María Galmarini, Miguel A Peñalva

(a)

(b)

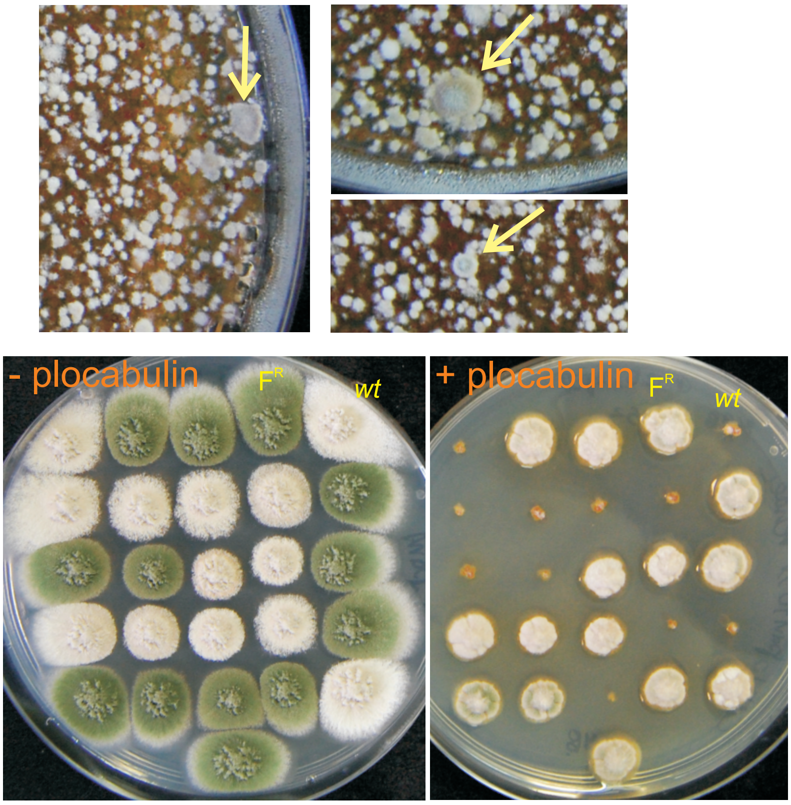

Segregation of resistance in backcrosses					
	Progeny obtained				
Mutant	Class A	Class C	Class D	Class E	Class F
Resistant	10	17	19	43	21
Sensitive	14	19	29	53	27
Comparison observed distribution with expected for one locus segregation					
Binomial test P-value (one-tailed)	0,2706	0,434	0,0967	0,1792	0,2354
Is discrepancy significant ?	no	no	no	no	no

Supplementary Fig. S1. Isolation of plocabulin-resistant *A. nidulans* mutants. Faster growing colonies (arrows) were selected over a background of micro-colonies arising after 10^6-10^8 viable UV-mutagenized spores were plated on 10 µM plocabulin and left in the dark at 37 °C for >4 days (a). After purification by serial spore dilutions, resistant strains were backcrossed to test whether resistance was segregating as a single genetic locus (b). In the upper panel of (b), n=24 progeny coming from a cross between the F^R parental (MAD4653) and a *wt* parental (MAD3688) were tested for growth on plates with or without plocabulin. In the lower panel, the binomial test *P*-values for the comparison between the numbers of progeny obtained and progeny expected if resistance were linked to one genetic locus showed that in all cases resistance segregated as a single Mendelian trait.

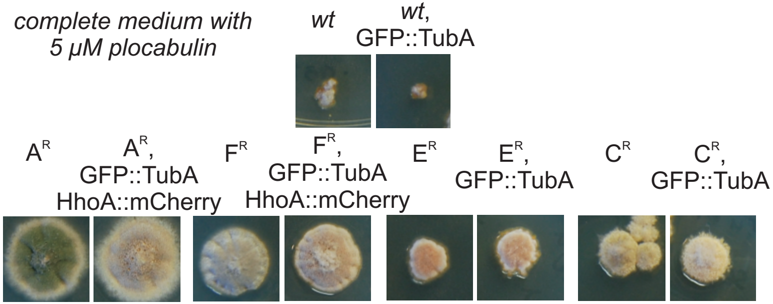

Supplementary Fig. S2 GFP-tagging of TubA does not have an effect on the resistance displayed by the mutants. Strains used: A^R (MAD5916 and MAD6141), F^R (MAD4751 and MAD6138), E^R (MAD6098 and MAD6149), C^R (MAD6095 and MAD5667), *wt* (MAD5321 and MAD4658).

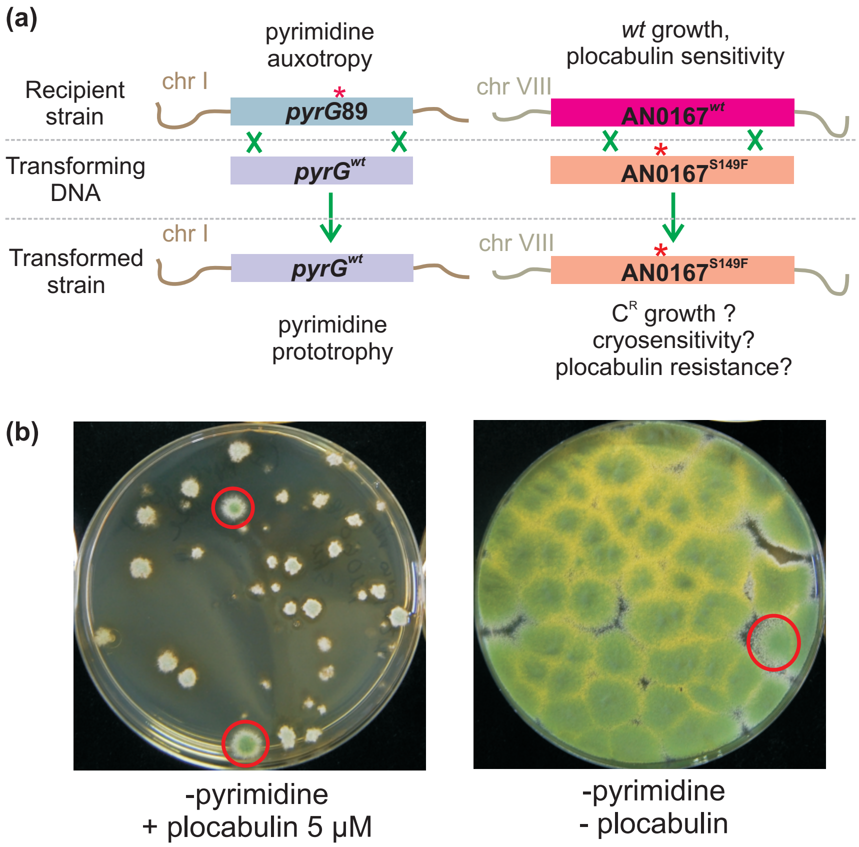

Supplementary Fig. S3. Reconstruction of *GCN3*-1 mutation by co-transformation using *pyrG*^{Anid} and AN0167 amplified from the C^{R} resistant. (a) Co-transformation strategy. (b) Transformation selective plates (see text for details). Encircled in red are colonies that have integrated the mutation in AN0167.

(a)

eIF2B alpha subunit

```
                          *        20         *        40         *        60         *
AN0167       1 : MTATDSQI-TKP-SEPFDIVATYNDLLRSDPDLTMPIAAIEALVLLLTHSPSSTISETLDLLEKSTTHLKKSI
Gcn3_Scer    1 : -------------MSEFNITETYLRFLEEDTEMTMPIAAIEALVTLLRIKTPETAAEMINTIKSSTEELIKSI
eIF2B1_Hom   1 : -------------MDDKELIEYFKSQMKEDPDMASAVAAIRTLLEFLKRDKGETIQGLRANLTSAIETLC-GV
EI2BA_SCHP   1 : MVESDSSGIVRHSQGEFDIVQVYKKFLQDDPEITMPVAAIEALVQLLSRSQAKTISEFMDILQNGSNTLKEGV

                      80         *       100         *       120         *       140
AN0167      72 : PNPIGLSAGTDLFQRYLITTLQRPGQLGPAGDFNAIRAHLLSNSRLFIRRAKESRDKIAGFGRGFVRDGSTVL
Gcn3_Scer   61 : PNSVSLRAGCDIFMRFVLRNLHL------YGDWENCKQHLIENGQLFVSRAKKSRNKIAEIGVDFIADDDIIL
eIF2B1_Hom  60 : DSSVAVSSGGELFLRFISLASLE------YSDYSKCKKIMIERGELFLRRISLSRNKIADLCHTFIKDGATIL
EI2BA_SCHP  74 : QNNISLSAGCDIFQRFVTRSLHD------VGDFEQCKRHLVENGKLFIQRARACRQRIAHLGYPLIRDGSVIL
```

S149F-AN0167

```
                    *       160         *       180         *       200         *       22
AN0167     145 : TNGGSRVVASLLQQAADEKGGPSAVRFNVIYVLSSPKGDIENPTAEPEGMETVRALRAKGVPVATIPESAVAY
Gcn3_Scer  128 : VHGYSRAVFSLLNHAANKFI-----RFRCV-VTES------RP--SKQGNQLYTLLEQKGIPVTLIVDSAVGA
eIF2B1_Hom 127 : THAYSRVVLRVLEAAVAAKK-----RFSVY-VTES------QP--DLSGKKMAKALCHLNVPVTVVLDAAVGY
EI2BA_SCHP 141 : THGFSRGVAAVLIAAAKRHV-----RFKVF-VTES------RP--SGSGCLMTRTLKNACIPTCMVLDSAVSF

                 0         *       240         *       260         *       280         *
AN0167     218 : SLGKADVVIVGAEGVVENGGIVSRMGTYQIGLLAKAMGKPFYVVAESHKFVRVYPLGQYDLPIEQHVIDFKTQ
Gcn3_Scer  187 : VIDKVDKVFVGAEGVAESGGIINLVGTYSVGVLAHNARKPFYVVTESHKFVRMFPLSSDDLPMAGPPLDFTRR
eIF2B1_Hom 186 : IMEKADLVIVGAEGVVENGGIINKIGTNQMAVCAKAQNKPFYVVAESFKFVRLFPLNQQDVPDK---FKYK--
EI2BA_SCHP 200 : TMNRVDLVLVGAEGVVENGGLINQIGTFQLAVFAKHAHKPFYAVAESHKFVRMFPLSQYDIPFSRPILEFDDP

                      300         *       320         *       340         *       360
AN0167     291 : EEVDNAKQ---QREPADASKSSGL---NTEFDAGSVDFTPPHLISALITDSGVLT-PSAVSEELIKIW-F
Gcn3_Scer  260 : TDDL-------------------E---DALR-GPTIDYTAQEYITALITDLGVLT-PSAVSEELIKMWYD
eIF2B1_Hom 254 : ADTL-------------KVAQTGQ---DLKEEHPWVDYTAPSLITLLFTDLGVLT-PSAVSDELIKLY-L
EI2BA_SCHP 273 : SPETVHPEPEPIPTPSDAIHNELIMNEEQIRNNPTLDVTPPEFVSGLITDLGIIDSKSGVSEELIKLY-L
```

(b)

eIF2B beta subunit

```
                          *        20         *        40         *        60         *
AN1344       1 : MPATSAPLTPGLASFLKSLKTNPIDTSIDNLISLLKRR-QIRHSRSCATATAYLLRSVISACRTSDASKLIERVQ
Gcd7_Scer    1 : MSSQAF------TSVHPNAATSDVNVTIDTFVAKLKRR-QVQGSYAIALETLQLLMRFISAARWNHVNDLIEQIR
eIF2B2_Hom   1 : MPGSAA-------------KGSELSERIESFVETLKRGGGPRSSEEMARETLGLLRQIITDHRWSNAGEIMELIR
EI2BB_SCHP   1 : MST--I-------------NVEHTYPAVSSLIADLKSR-KVQGPFAVAVETALVMRQVISQTRWSTVDQLIDTVR

                    80         *       100         *       120         *       140         *
AN1344      75 : SVGRRLIAAQPREMVVGNIVRRVLGLIRDEAEDDRDGDFT----LSDAGSESQPQTPRAGDEPSEFHGSDRGASK
Gcd7_Scer   69 : DLGNSLEKAHPTAFSCGNVIRRILAVLRDEVEED-----TMS-TTVTSTS-------------------------
eIF2B2_Hom  63 : REGRRMTAAQPSETTVGNMVRRVLKIIREEYGRLHGRSDESD---------------Q----QES----------
EI2BB_SCHP  60 : AVGSTLVKAQPTEFSCGNIIRRILRLIREEYQELLKTADENEKLIVSSSNSSSPSQKR----DIPSNEKLVQSHE

                        160         *       180         *       200         *       220
AN1344     146 : PISSLATHPVSMFSLLSHPEPETSLPGTPATGSPSGRLPGHTQNKDIRAEVLDGINEIIDELGQVDDQIAAYALD
Gcd7_Scer  113 : -VAEP--LISSMFNLLQKPEQPHQNRK-N--SSGSSS---MKTKTDYRQVAIQGIKDLIDEIKNIDEGIQQIAID
eIF2B2_Hom 109 : -----------LHKLLTSGGLNED---------------FSFHYAQLQSNIIEAINELLVELEGTMENIAACALE
EI2BB_SCHP 131 : PVSV--QMYSSMLNLLGRPTLESPTHS-K--TVGDS---RVTGGMDMRAVIISGIQDVIDELDKINTDIEVQSMD

                     *       240         *       260         *       280         *       300
AN1344     221 : HIHSNEIILTHT-SSTTVQKFLLKAA--AKRKFTVIHAESYPNNHEATHATVSGAASNDDEILSTESFQKPLIAH
Gcd7_Scer  179 : LIHDHEILLTPTPDSKTVLKFLITARERSNRTFTVLVTEGFPNNTKNAHE-----------------FAKKLAQH
eIF2B2_Hom 158 : HIHSNEVIMTIG-FSRTVEAFLKEAA--RKRKFHVIVAECAPF--CQGH-----------------EMAVNLSKA
EI2BB_SCHP 198 : HLHSNEIILTQG-CSKTVEAFLRFAA--KKRKFSVIVAEGFPNNQKGSH-----------------AMAKRIAQA

                          *       320         *       340         *       360         *
AN1344     293 : GITVILIPDSAVFALMSRVNKVILGTHSVLANGGLVA-AAGTRVIARAAKVHQTPCVVVSGVYKLSPVYPFDFES
Gcd7_Scer  237 : NIETLVVPDSAVFALMSRVGKVIIGTKAVFVNGGTISSNSGVSSVCECAREFRTPVFAVAGLYKLSPLYPFDVEK
eIF2B2_Hom 211 : GIETTVMTDAAIFAVMSRVNKVIIGTKTILANGALRA-VTGTHTLALAAKHHSTPLIVCAPMFKLSPQFPNEEDS
EI2BB_SCHP 253 : GIDTTVISDATIFAIMSRVNKVILGTHAILGNGGLVT-YSGAQLVAQAARHHATPVVVCSGIYKLSPVYPYDLES
```

Y417N-AN1344

```
                   380         *       400         *       420         *       440
AN1344     367 : LIEYGDSSKVIDYEDGDLVDQIDVQNPLYDYVFAELVDLYITNLGGHAPSYLYRIVSDHYRKEDI-SF----
Gcd7_Scer  312 : FVEFGGSQRIL--PRMDPRKRLDTVNQITDYVPPENIDIYITNVGGFNPSFIYRIAWDNYKQIDVHLDKNKA
eIF2B2_Hom 285 : FHKFVAPEEVLPFTEGDILEKVSVHCPVFDYVPPELITLFISNIGGNAPSYIYRLMSELYHPDDH-VL----
EI2BB_SCHP 327 : IIQLSSPDKIMSFNEGDLISRAEILNPYYDYIPPDLVDLFITNLGGYPPSYLYRIMNDTYDASDT-IL----
```

Supplementary Fig. S4 Alignment of eIF2Bα and eIF2Bβ from *A. nidulans*, *S. cerevisiae*, *H. sapiens*, *S. pombe*.

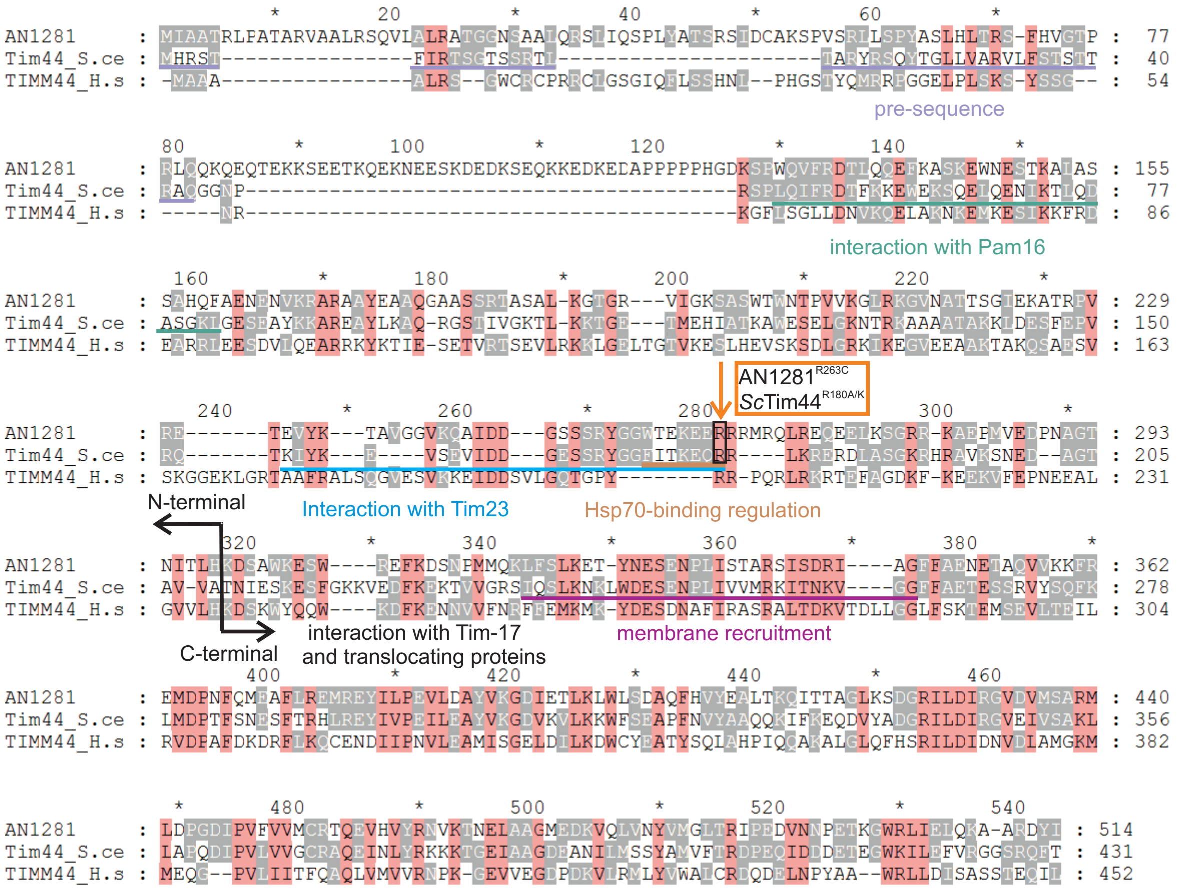

Supplementary Fig. S5. Alignment of Tim44 amino acid sequences. Alignment of *A. nidulans*, *S. cerevisiae* and *Homo sapiens* Tim44 amino acid sequences using webPRANK [1] (the sequences used are AspGD AN1281; TIM44_YEAST, accession Q01852 (UniProtKB); TIM44_HUMAN, accession O43615). Arg263 in *A. nidulans* TIM44 of which substitution by Cys confers plocabulin resistance is boxed. Substitution of the corresponding R180 in *S. cerevisiae* provokes mitochondrial protein import defects. Annotation of the function of the yeast protein domains is according to data in [2-6].

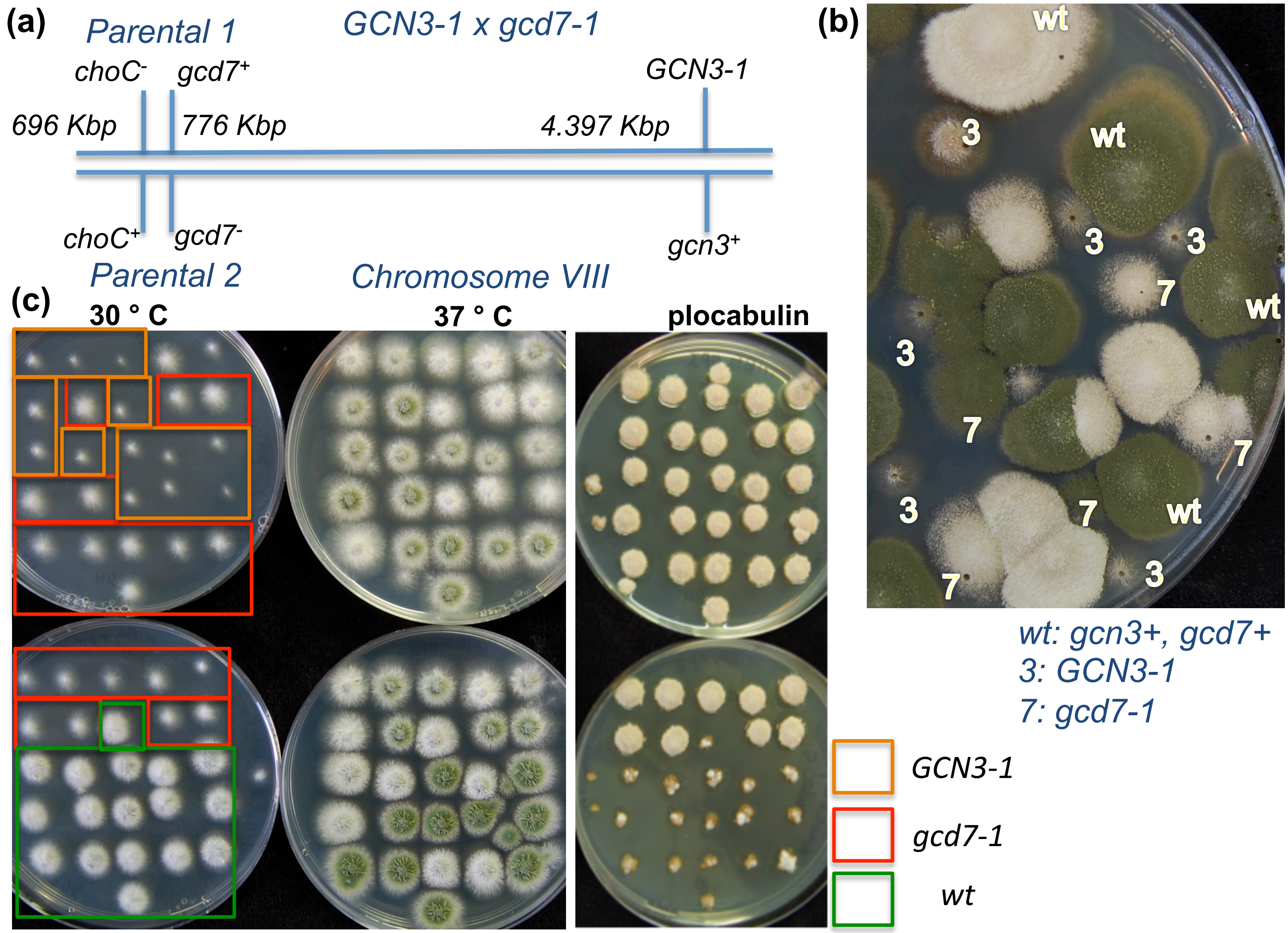

Supplementary Fig. S6. *GCN3*-1 *gcd7*-1 synthetic lethality. We crossed *GCN3*-1 (parental 1, *choC*-: choline auxotroph) and *gcd7*-1 (parental 2, *choC*+: choline prototroph) **(a)** and recovered three phenotypic classes **(b)** that were *wt*-, *GCN3*-1- and *gcd7*-1- like (roughly n= 40, 27, 24 respectively; P=0.092 in chi-square test comparing to the expected 1:1:1 ratio, not quite statistically significant; chi-square test at www.graphpad.com; please note that *GCN3*-1 are most probably underestimated because they grow much less than the *wt* from ascospores on complete media). We genotyped five *wt*–like, six *GCN3*-1–like and seven *gcd7*-1–like strains and confirmed that they carried one or the other or no mutation, but we did not recover any double mutant. Because *gcn3* / *gcd7* loci recombined freely (suggested by recovery of the *wt* strains at the expected 1/3 ratio), this indicates that the double *GCN*3-1 *gcd7*-1 mutant is unviable.

gcd7 is at about 80 Kbp distance from *choC* and the two genes are linked. In n=10 PCR-genotyped *gcn3+* progeny we obtained 1 *gcd7*/*choC* recombinant, while 3 *gcd7*/*choC* recombinants out of 48 progeny were scored based on phenotypic analyses [we can phenotypically differentiate *gcd7*-1 from *GCN3*-1, see **(b)** and (**c**)], suggesting that recombination between *gcd7* and *choC* is 5-10 %. *GCN3*-1 is roughly at 3.7 Mbps from *choC*+, suggesting the two genes are unlinked, in agreement with the free recombination observed between *gcd7* and *gcn3 loci* resulting in *wt* progeny (*gcd7* is between *choC* and *gcn3*). However, out of 13 *GCN3*-1 strains we only obtained 2 that were *choC*+ (*choC* / *gcn3* recombinants). Since *gcd7* and *choC* are linked, these strains would have much higher probability of being *gcd7*-1 rather than *gcd7*+. However, genotyping showed that *choC*+ *GCN3*-1 strains were *gcd7*+, thus originating from a recombination event within the short region between *gcd7* and *choC*. This buttressed the conclusion that the double *GCN3*-1 *gcd7*-1 mutant is unviable. To further strengthen this conclusion, we selected *choC*+ progeny by plating ascospores on synthetic complete without choline. From 48 *choC*+ progeny, the majority shows the *gcd7*-1 *gcn3*+ phenotype. 5 are *wt*-like, originating from two recombination events between *choC* / *gcd7* and *gcd7* / *gcn3*. Only 4 out of 48 *choC*+ strains are *GCN3*-1. Given that *gcd7* and *gcn3* recombine freely, this result is compatible with the *gcd7*-1 *GCN3*-1 double mutants being unviable.

Supplementary Table S1: Strains used in this work

Madrid (MAD) collection number	Genotype	Origin
**2**	biA1	Claudio Scazzocchio
**873**	pabaA1 yA2; ureB3 palB16	Herb Arst
**1426**	pyroA4 nkuAΔ::argB; riboB2	Berl Oakley
**1739**	pyrG89; pyroA4 nkuAΔ::bar	Herb Arst
**3685**	pabaA1; wA3	Herb Arst
**3688**	wA3; pantoB100	Herb Arst
**4629**	suA1ade20 yA2 ade20; acrA1; galA1; pyroA4; facA303; sB3; nicB8; riboB2	MSF/IBT26141 Clutterbuck Denmark/Glasgow 95
**4647**	A^R biA1	This work
**4648**	D^R biA1	This work
**4649**	B^R /(= $benA^{N100I}$) biA1	This work
**4650**	C^R biA1	This work
**4652**	E^R biA1	This work
**4653**	F^R biA1	This work
**4655**	fwA1 choC3 sD85	Herb Arst
**4659**	pyrG89; wA::$tubA^p$ ::gfp-tubA::$pyroA^{Af}$; pyroA4 nkuAΔ::argB; hhoA-mCherry::$pyrG^{Af}$; riboB2	Berl Oakley
**4736**	B^R pantoB100	This work
**4738**	A^R biA1; pantoB100	This work
**4740**	D^R biA1; wA3; pantoB100	This work
**4744**	C^R biA1; wA3; pantoB100	This work
**4745**	C^R pantoB100	This work
**4747**	E^R biA1; wA3; pantoB100	This work
**4751**	F^R biA1; wA3; pantoB100	This work
**4757**	B^R sD85 choC3	This work
**4852**	biA1; galG2; fwA1 facC307	FGSC
**4853**	pyroA4; pdhC2 cnxB11 fpaD43 chaA2	FGSC
**4855**	fwA1 pantoA10 sE15 $nirA^{c/d}$101	Herb Arst
**5319**	pyrG89; pyroA4 nkuAΔ::bar; riboB2	Herb Arst
**5321**	pyroA4 nkuAΔ::bar; riboB2	Herb Arst
**5386**	pyrG89; pyroA4 nkuAΔ::bar; gcn3Δ::$pyrG^{Af}$	This work
**5656**	biA1; wA3; $benA^{N100I}$ sD85 gcn3-1	This work
**5660**	biA1; choC3 gcn3-1	This work
**5736**	pyrG89; pyroA4 nkuAΔ::bar	Herb Arst
**5755**	pyrG89 tubCΔ::$pyrG^{Af}$; wA::$tubA^p$ ::gfp-tubA::$pyroA^{Af}$; pyroA4 nkuAΔ::bar; riboB2	This work
**5821**	wA::$tubA^p$ ::gfp-tubA::$pyroA^{Af}$; hhoA-mCherry::$pyrG^{Af}$; gcn3-1 (pyrG89?; pyroA4? nkuAΔ::argB?)	This work
**5877**	tubCΔ::pyrGAf; pyroA4; $benA^{N100I}$ riboB2 (pyrG89?; nkuAΔ::bar?)	This work
**5914**	$ploA^{Y223D}$; pyroA4 nkuAΔ::bar; riboB2	This work
**5916**	$ploA^{Y223D}$; pyroA4 nkuAΔ::bar; riboB2	This work
**5948**	biA1; pyroA4; pantoB100; gcd7-1 gcn3Δ:: $pyrG^{Af}$ (pyrG89?; nkuAΔ::bar?)	This work
**5950**	pyroA4; gcd7-1 (nkuAΔ::bar?)	This work
**5972**	pyrG89; ploAΔ::$pyrG^{Af}$; pyroA4 nkuAΔ::bar; riboB2	This work
**5991**	pyrG89; $cpcA^{GCN4}$Δ::$pyrG^{Af}$; pyroA4 nkuAΔ::bar; riboB2	This work
**6050**	biA1; wA3; choC3 $tim44^{R263C}$ (pantoB100?)	This work

**6079**	*pyrG89::pyrG*; *ploA*^{Y223D}; *pyroA4; nkuAΔ::bar*	This work
**6085**	*pyrG89*; *gcnEΔ*::*pyrGAf*; *pyroA4 nkuAΔ::bar*; *riboB2*	This work
**6087**	*gcnEΔ*::*pyrGAf*; *gcn3-1* (*biA? pyrG89?*; *pyroA4? nkuAΔ::bar?*; *riboB2? choC3?*)	This work
**6091**	*biA1*; *cpcA*^{GCN4}*Δ::pyrG*^{Af}; *pyroA4* (*nkuAΔ::bar?*)	This work
**6094**	*pyroA4 nkuAΔ::bar*; *riboB2*	This work
**6095**	*pyroA4 nkuAΔ::bar*; *riboB2 gcn3-1*	This work
**6098**	*pyroA4 nkuAΔ::bar*; *riboB2 tim44*^{R263C}	This work
**6106**	*pyrG89*; *ploA*^{Y223D}*-GFP*::*pyrG*^{Af}; *pyroA4 nkuAΔ::bar*	This work
**6107**	*pyrG89*; *ploA*^{Y223D}*-GFP*::*pyrG*^{Af} ; *pyroA4 nkuAΔ::bar*	This work
**6108**	*pyrG89*; *ploA-GFP*::*pyrG*^{Af}; *pyroA4 nkuAΔ::bar*	This work
**6109**	*pyrG89*; *ploA-GFP*::*pyrG*^{Af}; *pyroA4 nkuAΔ::bar*	This work
**6138**	*biA1; wA::tubA*^{p}*::gfp-tubA::pyroA*^{Af}; *ploF*^{I505N} (*pyroA4? nkuAΔ::argB?*; *hhoA-mCherry::pyrG*^{Af})	This work
**6142**	*pyrG89; ploA*^{Y223D}; *wA::tubA*^{p}*::gfp-tubA::pyroA*^{Af}; *pyroA4 nkuAΔ)*; *hhoA-mCherry::pyrG*^{Af}	This work
**6147**	*wA::tubA*^{p}*::gfp-tubA::pyroA*^{Af}; *hhoA-mCherry::pyrG*^{Af}; *tim44*^{R263C} (*pyrG89?*; *nkuAΔ::argB? pyroA4?)*	This work
**6230**	*wA::tubA*^{p}*::gfp-tubA::pyroA*^{Af}; *hhoA-mCherry::pyrG*^{Af}; (*pyrG89?*; *nkuAΔ::argB? pyroA4?)*	This work
**6444**	*pyrG89*; *ploF*^{I505N}::*pyrG*^{Af}; *pyroA4 nkuAΔ::bar*	This work
**6447**	*pyrG89*; *ploF*::*pyrG*^{Af}; *pyroA4 nkuAΔ::bar*	This work

Supplementary Table S2: DNA oligonucleotides used in this work

Primer identification number	Primer name	5´to 3´sequence
1	TubA Fw	CTTCTCGTTCTCCAATTTCTACCC
2	TubA Fw2	GCGTCTCTCTGTCGACTACGGC
3	TubA Rev	GCCAGCGTATCACCTAGACTACC
4	TubA Rev2	CCATGAAGCTGCAGTCGGAG
5	Fw BenA	CCAGTCTATCTTCATCCGACTTCG
6	BenA Fw2	GCCACCCTTTCCGTTCACC
7	Rev BenA	CCAGGGGTATTATTATGAGCTGATC
8	BenA Rev3	GGGTGCGCATGCAGATATCG
9	AP2014_18 Rev AN0167	GCAAATTCTCCGCGATGCC
10	AP2014_19 FWAN0167	CGTGCGAAAAGCCTGAGATGG
11	AP2014_22 Rev 5UTR GCN3	TGTGCTTAACGGGAGCTTATTAGC
12	AP2014_23 Fw 5UTR GCN3	CGCATACCTCCGCCATTAGTC
13	AP2014_24 FW 3UTR GCN3	TAAGTCCCTGGATGCACATATCG
14	AP2014_25 Rev 3UTR GCN3	CTTCTCAAGCAACTTGCGAATTG
15	AP2014_26 FW fus5GCN3pyrG	GCTAATAAGCTCCCGTTAAGCACAACCGGTCGC CTCAAACAATGCTC
16	AP2014_27 Rev fus3GCN3pyrG	CGATATGTGCATCCAGGGACTTAGTCTGAGAGG AGGCACTGATGCG
17	AP2016_41 FWextGCN3	GTTGATACTCGCGTCGCATGG
18	AP2016_42 RevextGCN3	GCTTCAATGCAGGCGCAAC
19	AP2015_1 FWAN1344 GCD7	CGCATGTCGCCCGCCCTG
20	AP2015_2 RevAN1344 GCD7	CACGCTGGCGTGCATAATCC
21	AP2015_3 FWAN6864 eIF2Bdelta	CTCCAATGCCCCTCCTGATTG
22	AP2015_4 RevAN6864 eIF2Bdelta	GAGCGGCGCAAGAGACAAGG
23	AP2015_5 RevORFAN6864	GGCTGCTGACCCCGCTGC
24	AP2016_19 FWbefstopAN3969	CGTCGCGCATATGATGGAGAC
25	AP2016_20 Rev3UTRAN3969	GCGAAGAGGGCGACAGACG
26	AP2016_22 RevAN3969pyrG	CATGGTCTCCATCATATGCGCGACGCTGTCTGAG AGGAGGCACTGATGCG
27	AP2016_43 FW 5UTR AN3969	CCGGTAGGTCAGTGAGAATGGG
28	AP2016_44 Rev 5UTR AN3969	CTCTGGGGCGCGATGGAG
29	AP2016_45	CGCTCTCCATCGCGCCCCAGAGACCGGTCGCCT

	fus5AN3969pyrG	CAAACAATGCTCTTC
30	AP2016_7 FWorfAN4084	CCCAGAGCAAGGTCTCCACG
31	AP2016_8 RevAN4084stop	GACTCATTGCAGATTGTGAGAGGC
32	AP2016_23 FWan3969orf	CCTCAGCACAGCCCCAGCC
33	AP2016_24 Rev AN3969 ORF	GCACGCTGCTAACATATCCGC
34	AP2016_15 FWORF AN8292	GCCAGGCAGTCTTACTTC
35	AP2016_16 RevORF N8292	GGTCGTGGAGAAGAAGATCCG
36	AP2016_36 FwcDNAAN3969	CACTCGCTCTCCATCGCGC
37	AP2016_38 FWcDNAAN3969	GCCGAACGCTGGCTTGATG
38	AP2016_40 RevcDNAAN3969	GAGCAGAGGAATAAACTGGTGTAGG
39	AP2016_76 Fw ORF AN3969	AGGCGAGTGGGCTGTGAAC
40	AP2016_80 RevcDNAAN3969	CAATAGGTAGACAATGGTTGTCTATGAG
41	AP2015_27 FW5UTRTubC	CGGATCATCCATGGTTCCCG
42	AP2015_28 Rev5UTRTubC	GATGGGCTTGAAGGGATGGG
43	AP2015_29 FW3UTRTubC	GGAGTAGTGGCCTCCTTTCTTGG
44	AP2015_30 Rev3UTRTubC	CCGGTAAACGCGCAACGC
45	AP2015_31 FWTubCpyrGfus	CAAAAACCCATCCCTTCAAGCCCATCACCGGTC GCCTCAAACAATGCTCTTC
46	AP2015_32 RevTubCpyrGfus	TAACCAAGAAAGGAGGCCACTACTCCCTGTCTG AGAGGAGGCACTGATGCG
47	AP2016_48 FWAN1281	GTGCGGCGTCTTCACGAAC
48	AP2016_49 RevAN1281	GGAACTGAGCGTCTGAGAGCC
49	AP2016_46 FWclassEAN1360	GGGCAGATGGTCTGCACGG
50	AP2016_47 Rev classEAN1360	GCAGCACTCGCAGCCGAC
51	AP2016_51 FwAN5786	GGTTGCGTGTGACTCGCG
52	AP2016_52 revAN5786	CTCACCTTGCGCGAACACG
53	AP2016_71 FWclFcandAN8345	GCGGCGAAGGACATCTGC
54	AP2016_72 RevclFcandAN8345	CCGAGCCGTACTTGGTCGG
55	AP2016_78 FWAN12394 clF	CCCAGTCTTACGGGTGCCAC
56	AP2016_79 RevAN12394 chrV	CAGACGGACCATCGAGATGC
57	AP2016_81 Rev ORF	AGTAGCTTGACAAACTGATATCATGGTC

58	AP2016_83 Fw 3UTR	TAGATAACTCATAGACAACCATTGTCTACC
59	AP2016_82 FusAN3969CtermGFP	GGAGACCATGATATCAGTTTGTCAAGCTACTGG AGCTGGTGCAGGCGCTGGAGCC
60	AP2016_84 Revfus·utr3969pyrG	GGTAGACAATGGTTGTCTATGAGTTATCTAGTCT GAGAGGAGGCACTGATGCG
61	AP2016_39 RevCDNAAN3969	CGCGGAGAGTACAGGGAAGAG
62	AP2016_86 Fw AN8345	GGCATGTCGGCGTGCAAG
63	AP2016_91 Rev8345	GTAAGAAGATCATCCAAGCTGTTTACC
64	AP2016_94 FW3utr8345	TTTTCGTCATGCTGAGGATGCAC
65	AP2016_95 Rev3utr8345	GGGATTCTGGCGTCGATGATC
66	AP2016_92 FWFus8345pyrG	CAGGTAAACAGCTTGGATGATCTTCTTACACCG GTCGCCTCAAACAATGCTCTTC
67	AP2016_93 Revfus8345pyrG	GTGCATCCTCAGCATGACGAAAACTGTCTGAGA GGAGGCACTGATGCG
68	AP2016_59 FW5primeGcn4	CTCCACTGTGCGGCAGACC
69	AP2016_60 Rev 5primeGcn4	CAGGGAGGAAAGCCAGGCAG
70	AP2016_61 Fw3primeGcn4	GAAGCTCTTCCGTTGTGGCG
71	AP2016_62 Rev3primeGcn4	GGTGTGCTGGCTCCATGGC
72	AP2016_63 Fw5gcn4pyrG	CGCTGCCTGGCTTTCCTCCCTGACCGGTCGCCTC AAACAATGCTCTTC
73	AP2016_64 Rev3gcn4pyrG	CCGCCACAACGGAAGAGCTTCCTGTCTGAGAGG AGGCACTGATGCG
74	AP2016_53 Fw5UTRGcnE	CGTAGACTCCAACGGCACAGATC
75	AP2016_54 Rev5UTRGcnE	GCTTGTTGCTATGCGAGTAAGC
76	AP2016_55 FW3UTRGcnE	CGATATGAGGCGTTGAATGGG
77	AP2016_56 Rev3UTRGcnE	GAAAGCGAGAGGCTGACGAGC
78	AP2016_57 FWfus5gcnEpyrG	CAGCTTACTCGCATAGCAACAAGCACCGGTCGC CTCAAACAATGCTCTTC
79	AP2016_58 Revfus3gcnEpyrG	CTCCCATTCAACGCCTCATATCGCTGTCTGAGAG GAGGCACTGATGCG
80	AP2016_73 Fw5UTRGcnE	CGGACAATTTCAGCTTACTCGC
81	AP2016_74 Rev3UTRGcnE	CCCATTCAACGCCTCATATCG
82	MAPS125 pyrGsequp	CAGCCATCCCACTTCCAGCTTC
83	MAPS126 pyrGseqdw	CTGGTAATACTATGCTGGCTGC
84	AP2016_85 fw an1281	CTCTGCCGCTTTACAACGGTC

Supplementary Table S3. Polymorphisms/insertions/deletions within the *sE*/*nirA* genomic region of C^R

Gene	Position	Mutation
**AN0170 – *sE* (*trxA*)**	4,388,838	
AN0167 – *gcn3*	4,397,716 / 7	TCT>TTC Ser149Phe
AN0149 – *mdpF*	4,450,754	delT (intron)
-	4,534,885	delG
AN10019 – *oca2*/*HRK1*	≈4,557,560-4,557,570	Deletion part of intron
**AN0098 – *nirA***	4,617,254	

Supplementary Table S4. Resistance-causative candidate single *nt* polymorphisms on E[R] chromosome VIII

Code	Gene<br>*Homologues-function*	Position	Mutation
**i**	AN9304 (5´UTR)<br>*TEF4- Gamma subunit of the translation elongation factor eEF1B*	231.379	(G>A)
**ii**	AN9348<br>*Putative aryl-alcohol oxidase-related protein*	373.693	(T>C) Asn551Asp
**iii**	AN1359 (5´UTR)<br>*Axl2-cell polarity protein-axial budding*	743.175	(T>C)
**iv**	AN1281<br>*TIM44-mitochondrial translocase component*	962.918	(G>A) Arg263Cys
**v**	AN0541<br>*CTS2-putative chitinase*	3.241.807	(T>A) Asn1319Lys

Supplementary Table S5. A^R resistance causing candidate SNPs on chromosome II

Gene	Position	Mutation
AN8292 (XPG-protein family related domain, similarity to *S. pombe* Ast1 nuclease)	1.407.484	(C>T) Gln111stop
AN4084 (glycosyltrasferase family-specific domain)	2.148.128	(C>T) Ser475Leu
AN3969 (Zn2-Cys6 DNA-binding domain)	2.475.691	(A>C) Tyr235Asp

Supplementary Table S6. F^R resistance-causing candidate SNPs on F^R chromosome V

Gene	Position	Mutation
AN8345 (Zn2-Cys6 DNA-binding domain)	187.929	T>A Ile505Asn (after correcting intron annotation)
AN12394 (unknown function)	760.829	(T>G) intron
AN5786 (homologue Mdm31-mitochondrial protein)	1.708.844 1.708.845	(GG>AA) Thr152Ile

S1 Movie. Mitosis in the tip cell of *wt* hyphae in the presence or absence of plocabulin. Comparative progression of mitosis in a *wt* strain before (left panel) and 25 min after the addition of plocabulin in the growing medium (right panel). Elapsed time is the same for both movies (in min:sec:msec). Note that without the drug mitosis progresses through anaphase, while in the presence of plocabulin spindle elongation appears blocked and nuclei are blocked in metaphase. Frames are merge of maximal projections of z-stacks acquired for the red or green channel with the multidimensional acquisition function of Metamorph.

S2 Movie. Mitosis in the tip cell of an F^R hypha on plocabulin. Note the almost synchronous progression through the anaphase of all nuclei in the tip cell except from the leading nucleus (closest to the growing apex, towards the right edge of the micrograph) that appears blocked in metaphase. This is in agreement with MTs in F^R being resistant to plocabulin at posterior positions of the tip cell, while they depolymerize close to the apex (see Fig 4). Elapsed time in min:sec:msec. Frames are merge of maximal projections of z-stacks acquired for the red or green channel.

References

1 Loytynoja, A. & Goldman, N. webPRANK: a phylogeny-aware multiple sequence aligner with interactive alignment browser. *BMC bioinformatics* **11**, 579, doi:10.1186/1471-2105-11-579 (2010).

2 Banerjee, R., Gladkova, C., Mapa, K., Witte, G. & Mokranjac, D. Protein translocation channel of mitochondrial inner membrane and matrix-exposed import motor communicate via two-domain coupling protein. *eLife* **4**, e11897, doi:10.7554/eLife.11897 (2015).

3 Schiller, D., Cheng, Y. C., Liu, Q., Walter, W. & Craig, E. A. Residues of Tim44 involved in both association with the translocon of the inner mitochondrial membrane and regulation of mitochondrial Hsp70 tethering. *Molecular and cellular biology* **28**, 4424-4433, doi:10.1128/mcb.00007-08 (2008).

4 Schilke, B. A., Hayashi, M. & Craig, E. A. Genetic analysis of complex interactions among components of the mitochondrial import motor and translocon in Saccharomyces cerevisiae. *Genetics* **190**, 1341-1353, doi:10.1534/genetics.112.138743 (2012).

5 Marom, M. *et al.* Interaction of the Tim44 C-terminal domain with negatively charged phospholipids. *Biochemistry* **48**, 11185-11195, doi:10.1021/bi900998v (2009).

6 Ting, S. Y., Schilke, B. A., Hayashi, M. & Craig, E. A. Architecture of the TIM23 inner mitochondrial translocon and interactions with the matrix import motor. *The Journal of biological chemistry* **289**, 28689-28696, doi:10.1074/jbc.M114.588152 (2014).
